# Supplementary material for: Comparative analysis of the impact of chickenpox and herpes zoster vaccination in Belgium under two different exogenous boosting mechanisms
Source: PLoS One. 2026 Mar 19;21(3):e0342896. doi: 10.1371/journal.pone.0342896 (PMC13001986; doi:10.1371/journal.pone.0342896)
Supplement: S1 File — (PDF) [file pone.0342896.s001.pdf]

# S1 file for: "Comparative analysis of the impact of chickenpox and herpes zoster vaccination in Belgium under two different exogenous boosting mechanisms"

James Wambua[1*], John C. Lang[2], Benson Ogunjimi[3,4,5], Niel Hens[1,3], Philippe Beutels[3,6]

February 10, 2026

[1]Data Science Institute, I-BioStat, Hasselt University, Hasselt, Belgium.
[2] Merck Canada Inc., Kirkland, Quebec, Canada.
[3]Centre for Health Economics Research and Modelling of Infectious Diseases, Vaccine & Infectious Disease Institute, University of Antwerp, Antwerp, Belgium.
[4] Antwerp Unit for Data Analysis and Computation in Immunology and Sequencing (AUDACIS), University of Antwerp, Antwerp, Belgium.
[5]Department of Paediatrics, Antwerp University Hospital, Antwerp, Belgium.
[6] School of Public Health and Community Medicine, The University of New South Wales, Sydney, Australia.
* Corresponding author: james.wambua@uhasselt.be

## Supporting information

# 1 UA compartmental model

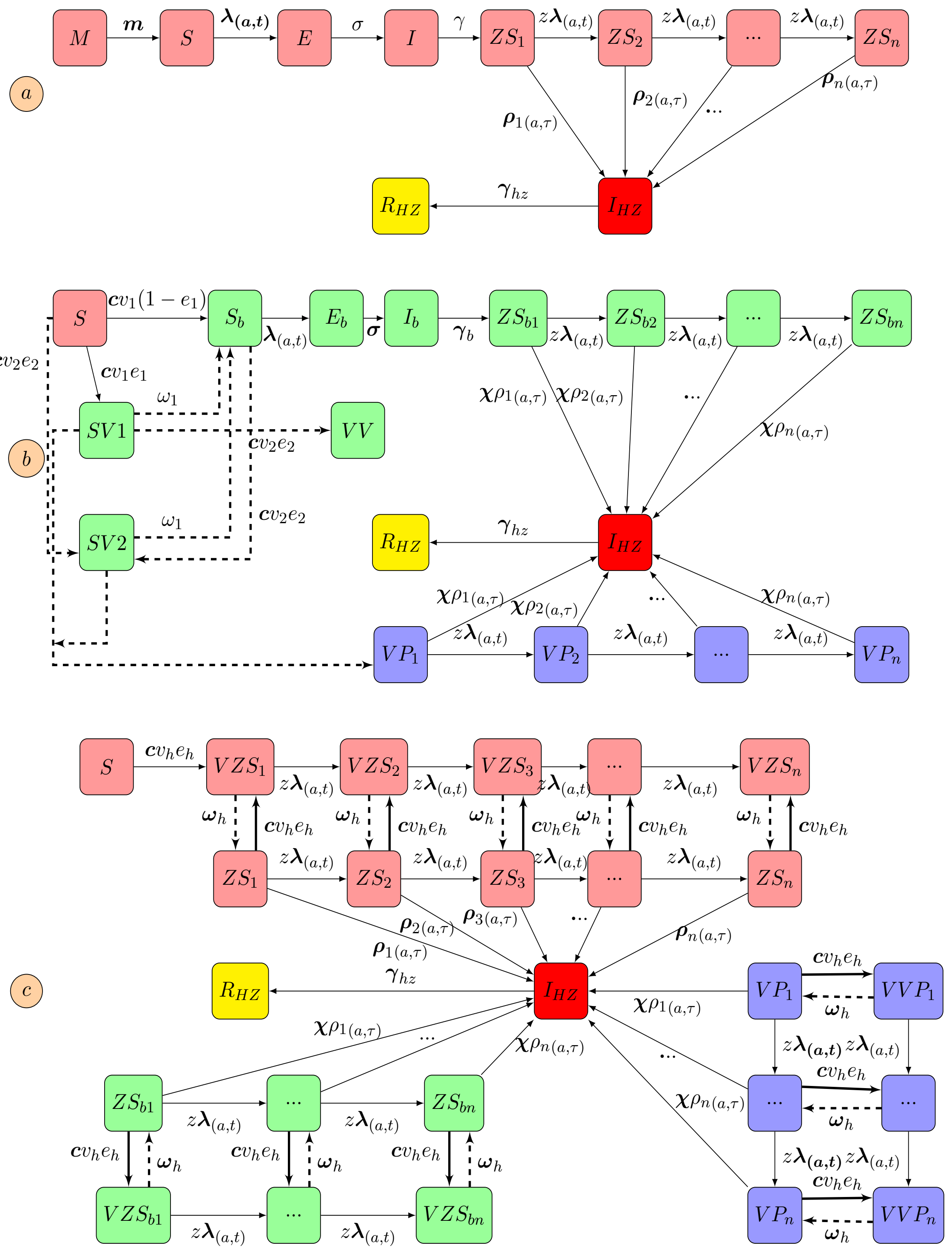


Supplementary Fig 1: Representation of compartmental model structure with 100 one year age groups: **(a)** Compartments representing the natural history of VZV and its subsequent reactivation to HZ. $M$: Maternally immune. $S$: susceptible to VZV (wild-type). $E$: Exposed (wild-type). $I$: Infected (wild-type). $ZS_i$: Susceptible to HZ who have **i** episodes of boosting (**(i=1,2,...,5)**), $I_{HZ}$: HZ infected, $R_{HZ}$: HZ recovered. **(b)** Model including CP vaccination. $S_b$: Susceptible to breakthrough CP, $E_b$: Exposed to breakthrough CP,$I_b$: Infected by breakthrough CP, $ZS_{bi}$: HZ susceptible from breakthrough CP who have **i** episodes of boosting, $SV_i$: CP vaccinated with first and second doses, $VP_i$: CP vaccinated and HZ susceptible who have **i** episodes of boosting. **(c)** Model of HZ vaccination and HZ reactivation from the vaccine strain. $VZS_i$: Individuals infected with wild-type CP and vaccinated against HZ who have **i** episodes of boosting, $VZS_{bi}$: Individuals infected with breakthrough CP and vaccinated against HZ who have **i** episodes of boosting, $VVP_i$: Susceptible to HZ reactivation from the vaccine strain and vaccinated against HZ.

## 1.1 UA model structure for CP and HZ

The UA team developed a discrete deterministic compartmental model to explore the VZV transmission dynamics and HZ reactivation in Belgium. In the model, newborns are protected by maternal antibodies (compartment $\boldsymbol{M}$) for their first 6 months on average after which they instantaneously become susceptible to VZV (i.e, wild-type) infection (compartment $\boldsymbol{S}$). Susceptible individuals are exposed to VZV through an age and time-dependent force of infection (FOI) $\lambda(a,t)$ and move to the latent compartment ($\boldsymbol{E}$), see Subsection 1.3 for the FOI computation. After a latent period, the exposed individuals become infectious and move to the infectious compartment ($\boldsymbol{I}$) at rate $\sigma$. Afterwards, individuals recover at rate $\gamma$ and acquire long-lasting immunity against VZV. After recovery from CP, individuals enter into stages of HZ susceptibility ($\boldsymbol{ZS_i}$). We considered 5 HZ susceptibility stages. This was informed by exploratory modeling as well as previous CP and HZ models [1]. The risk of HZ reactivation is modeled using the progressive immunity boosting mechanism as described in Subsection 2.1 in the main text. In this formulation, and with each immunological boosting event, individuals shift in sequence of classes with boosted immunity. Individuals are exogenously boosted at a force of boosting (FOB) $z\lambda(a,t)$, where $z$ is the boosting intensity. Thus, HZ susceptible individuals in the model can either develop HZ and acquire long term immunity to HZ after recovery or can be boosted by VZV exposure and moved to the next HZ susceptibility stage with time since last exposure $\tau = 0$. More detailed description of the compartments is contained in Supplementary Table 1.

The population level age-structured compartmental model consists of 100, 1-year age classes spanning from 0 to 99 years of age. Demographic changes in the model were modeled by implementing age-specific mortality rates from STATBEL (Statistics Belgium) [2] and age-specific fertility rate predictions of the Belgian population from the United Nations world population prospects [3]. The model considered constant age-specific migration schedules which were modeled using the Rogers and Castro model [4, 5] using the Belgian migration data from STATBEL. We assumed that migration in the model depended on the age-specific population profiles in each compartment. This assumption was taken because the CP sero-prevalence data and data pertaining immunized immigrants against CP and HZ are lacking in the literature. The inflow of births and aging in the model takes place in the beginning of the year where individuals belonging to age class $(a, a = 0, ..., 99)$ shift to age class $(a + 1)$ for CP compartments. While for the HZ susceptibility compartments, individuals are shifted from age class $(a, \tau)$ to $(a + 1, \tau + 1)$, where $\tau$ is time since last CP exposure in years. In each time step in the model, HZ susceptibility individuals who are re-exposed to CP are shifted to the next HZ susceptibility stage with $\tau = 0$.

The model consists of three main components to represent the transmission dynamics of CP and its subsequent reactivation to HZ as well as inco-operates the effect of both CP and HZ vaccination. The dynamical model structure for the natural history of CP and HZ under the progressive immunity boosting mechanism is depicted in (Supplementary Figure 1**a**).
Both the CP and HZ vaccination programs are assumed to begin in the year 2023. Children are offered the first dose of CP at age ($a_1$), at coverage of ($cv_1$), and vaccine efficacy of ($e_1$). Among the vaccinated, a fraction (1-$e_1$) becomes susceptible to breakthrough infection (compartment $\boldsymbol{S_b}$). Whilst the fraction ($e_1$) is fully protected (compartment $\boldsymbol{SV_1}$). The protection conferred by the vaccine is assumed to wane at a rate ($\omega_1$) making individuals to become susceptible to breakthrough infection which they acquire at the same FOI as fully susceptible individuals.

Breakthrough cases are assumed to be 50% contagious, compared to wild-type VZV cases [6]. The second dose of CP is offered to individuals who are susceptible to either wild-type or breakthrough VZV at age ($a_2$), coverage ($cv_2$) and efficacy ($e_2$) taken to be equal to ($e_1$). We assume the second dose provides full and permanent protection (compartment $\boldsymbol{VV}$) to all individuals who successfully got immunized by the first dose and are still immune at age ($a_2$). Individuals who become susceptible to breakthrough VZV after the first dose, enter into a compartment ($\boldsymbol{SV_2}$) of full short-term protection which wanes at rate ($\omega_1$) to compartment ($\boldsymbol{S_b}$). Individuals who experienced breakthrough VZV infection can either be boosted at the same FOB ($z\lambda(a,t)$) or can develop HZ at a reactivation risk $\chi\rho_i(a,\tau)$ where $\chi$ is an attenuation coefficient representing the relative HZ susceptibility in those individuals who were vaccinated with respect to unvaccinated individuals [7]. Individuals vaccinated against CP are later exposed to VZV vaccine strain reactivation at the same reduced factor $\chi$ as compared to those who experienced wild-type VZV (Supplementary Figure 1**b**). Individuals who received the first dose of the CP vaccine move to the first HZ susceptibility compartment $\boldsymbol{VP_1}$. The second dose of the CP vaccine is assumed to act as a boosting event in the model, at which individuals shift to the next HZ susceptibility compartment $\boldsymbol{VP_i}$.

HZ vaccination is offered at age ($a_h$) with a coverage ($cv_h$) and efficacy ($e_h$) to individuals who are completely susceptible to CP, who experienced either wild-type ($\boldsymbol{ZS_i}$) or breakthrough VZV ($\boldsymbol{ZS_{bi}}$) and those who got vaccinated against CP ($\boldsymbol{VP_i}$). HZ vaccinated individuals can either be boosted at the same FOB $z\lambda(a,t)$ or can loose immunity at an average rate of ($\omega_h$) and revert to HZ susceptibility compartments corresponding to their previous VP exposures accordingly (Supplementary Figure 1**c**).

The equations governing the model are formulated in discrete time and are fully presented in Subsection 1.2. The epidemiological parameter values for this compartmental model are contained in Supplementary Table 2.

## 1.2 Full equations of the compartmental model

$$
\begin{aligned}
S(a,t+\Delta t) &= S(a,t) - (\lambda(a,t) + \mu(a) + cv_1e_1 + cv_1(1-e_1) + cv_2e_2p_2)S(a,t)\Delta t \\
E(a,t+\Delta t) &= E(a,t) + (\lambda(a,t)S(a,t) - \sigma E(a,t) - \mu(a)E(a,t))\Delta t \\
I(a,t+\Delta t) &= I(a,t) + (\sigma E(a,t) - \gamma I(a,t) - \mu(a)I(a,t))\Delta t \\
ZS_i(a,\tau,t+\Delta t) &= ZS_i(a,\tau,t) - (z\lambda(a,t)ZS_i(a,\tau,t) - \rho_i(a,\tau)ZS_i(a,\tau,t) - cv_he_hZS_i(a,\tau,t) + \\
&\quad \omega_hVZS_i(a,\tau,t) - \mu(a)ZS_i(a,\tau,t))\Delta t \\
VZS_i(a,\tau,t+\Delta t) &= VZS_i(a,\tau,t) + (cv_he_hZS_i(a,\tau,t) - \omega_hVZS_i(a,\tau,t) - \mu(a)VZS_i(a,\tau,t) - \\
&\quad z\lambda(a,t)VZS_i(a,\tau,t))\Delta t \\
S_b(a,t+\Delta t) &= S_b(a,t) + (cv_1(1-e_1)S(a,t) + \omega_1SV_1(a,t) + \omega_2SV_2(a,t) - cv_2e_2S_b(a,t) - \\
&\quad (\lambda(a,t) + \mu(a))S_b(a,t))\Delta t \\
SV_1(a,t+\Delta t) &= SV_1(a,t) + (cv_1e_1S(a,t) - \omega_1SV_1(a,t) - cv_2e_2SV_1(a,t) - \mu(a)SV_1(a,t))\Delta t \\
SV_2(a,t+\Delta t) &= SV_2(a,t) + (cv_2e_2S(a,t) + cv_2e_2S_b(a,t) - \omega_2SV_2(a,t) - \mu(a)SV_2(a,t))\Delta t \\
VV(a,t+\Delta t) &= VV(a,t) + (cv_2e_2SV_1(a,t) - \mu(a)VV(a,t))\Delta t \\
E_b(a,t+\Delta t) &= E_b(a,t) + (\lambda(a,t)S_b(a,t) - \sigma E_b(a,t) - \mu(a)E_b(a,t))\Delta t \\
I_b(a,t+\Delta t) &= I_b(a,t) + (\sigma E_b(a,t) - \gamma I_b(a,t) - \mu(a)I_b(a,t))\Delta t \\
ZS_{bi}(a,\tau,t+\Delta t) &= ZS_{bi}(a,\tau,t) - (z\lambda(a,t)ZS_{bi}(a,\tau,t) - \chi\rho_i(a,\tau)ZS_{bi}(a,\tau,t) - cv_he_hZS_{bi}(a,\tau,t) + \\
&\quad \omega_hVZS_{bi}(a,\tau,t) - \mu(a)ZS_{bi}(a,\tau,t))\Delta t \\
VZS_{bi}(a,\tau,t+\Delta t) &= VZS_{bi}(a,\tau,t) + (cv_he_hZS_{bi}(a,\tau,t) - \omega_hVZS_{bi}(a,\tau,t) - z\lambda(a,t)VZS_{bi}(a,\tau,t) - \\
&\quad \mu(a)VZS_{bi}(a,\tau,t))\Delta t \\
VP_i(a,\tau,t+\Delta t) &= VP_i(a,\tau,t) - (z\lambda(a,t)VP_i(a,\tau,t) - \chi\rho_i(a,\tau)VP_i(a,\tau,t) - cv_he_hVP_i(a,\tau,t) + \\
&\quad \omega_hVVP_i(a,\tau,t) - \mu(a)VP_i(a,\tau,t))\Delta t \\
VVP_i(a,\tau,t+\Delta t) &= VVP_i(a,\tau,t) + (cv_he_hVP_i(a,\tau,t) - \omega_hVVP_i(a,\tau,t) - z\lambda(a,t)VVP_i(a,\tau,t) - \\
&\quad \mu(a)VVP_i(a,\tau,t))\Delta t \\
I_{HZ}(a,t+\Delta t) &= I_{HZ}(a,t) + (\sum_i \rho_i(a,\tau)ZS_i(a,\tau,t) + \sum_i \chi\rho_i(a,\tau)ZS_{bi}(a,\tau,t) + \sum_i \chi\rho_i(a,\tau)VP_i(a,\tau,t) - \\
&\quad \gamma_{hz}I_{HZ}(a,t) - \mu(a)I_{HZ}(a,t))\Delta t \\
R_{HZ}(a,t+\Delta t) &= R_{HZ}(a,t) + (\gamma_{hz}I_{HZ}(a,t) - \mu(a)R_{HZ}(a,t))\Delta t
\end{aligned}
$$

### Boundary conditions

$$ZS_1(a,0,t+\Delta t) = (\gamma I(a,t))\Delta t$$
$$ZS_{i,i=2,3,4,5}(a,0,t+\Delta t) = (z\lambda(a,t)\sum_{\tau} ZS_{i,i=2,3,4,5}(a,\tau,t))\Delta t$$
$$ZS_{b1}(a,0,t+\Delta t) = (\gamma_b I_b(a,t))\Delta t$$
$$ZS_{bi}(a,0,t+\Delta t) = (z\lambda(a,t)\sum_{\tau} ZS_{bi,i=2,3,4,5}(a,\tau,t)\Delta t$$
$$VP_1(a,0,t+\Delta t) = (cv_1 e_1 S(a,t) + cv_2 e_2 S_b(a,t))\Delta t$$
$$VP_{i,i=2,3,4,5}(a,0,t+\Delta t) = (z\lambda(a,t)\sum_{\tau} VP_{i=2,3,4,5}(a,\tau,t))\Delta t$$
$$VZS_{i=2,3,4,5}(a,0,t+\Delta t) = (z\lambda(a,t)\sum_{\tau} VZS_{i,i=2,3,4,5}(a,\tau,t))\Delta t$$
$$VZS_{bi,i=2,3,4,5}(a,0,t+\Delta t) = (z\lambda(a,t)\sum_{\tau} VZS_{bi,i=2,3,4,5}(a,\tau,t))\Delta t$$
$$VVP_{i,i=2,3,4,5}(a,0,t+\Delta t) = (z\lambda(a,t)\sum_{\tau} VVP_{i,i=2,3,4,5}(a,\tau,t))\Delta t$$

| Compartment | Description of the compartment |
|---|---|
| $M(a,t)$ | Newborns protected by maternal antibodies |
| $S(a,t)$ | Individuals susceptible to CP infection |
| $E(a,t)$ | Individuals exposed to CP (wild-type) |
| $I(a,t)$ | Individuals infected with CP (wild-type) |
| $ZS_i(a,\tau,t)$ | Individuals recovered from CP and susceptible to HZ with $i$ boosting episodes |
| $I_{HZ}(a,t)$ | Individuals infected with HZ from wild type VZV reactivation |
| $R_{HZ}(a,t)$ | Individuals recovered from HZ |
| $S_b(a,t)$ | Vaccine failures from CP vaccination and susceptible to breakthrough infection |
| $E_b(a,t)$ | Individuals exposed to breakthrough CP |
| $I_b(a,t)$ | Individuals infected with breakthrough CP |
| $ZS_{bi}(a,\tau,t)$ | Individuals recovered from breakthrough CP and susceptible to HZ with $i$ episodes of boosting |
| $SV_1(a,t)$ | Individuals vaccinated with the first dose of CP vaccine |
| $SV_2(a,t)$ | Individuals vaccinated with the second dose of CP vaccine only |
| $VV(a,t)$ | Individuals vaccinated with both doses of CP vaccines |
| $VP_i(a,\tau,t)$ | Individuals vaccinated against CP and who are susceptible to HZ reactivation with $i$ episodes of boosting |
| $VZS_i(a,\tau,t)$ | Individuals susceptible to HZ reactivation from wild-type CP and vaccinated against HZ, with $i$ episodes of boosting |
| $VZS_{bi}(a,\tau,t)$ | Individuals susceptible to HZ reactivation from breakthrough CP and vaccinated against HZ, with $i$ episodes of boosting |
| $VVP_i(a,\tau,t)$ | Individuals susceptible to HZ reactivation from vaccine strain and vaccinated against HZ |

Supplementary Table 1: Symbols of the description of the compartments of the mathematical model. All compartments are stratified by age $a$. In addition, the compartments for the HZ susceptible are further stratified with respect to the time elapsed since the last CP exposure event, $\tau$.

### 1.3 Force of infection

The transmission dynamics of VZV is governed by an age- and time-dependent force of infection (FOI)$\lambda(a,t)$ defined as the instantaneous rate at which a susceptible individual in age class $(a)$ acquires infection at time $(t)$.

$$\lambda(a,t) = \beta \sum_{a^{'}=0} C(a,a^{'})[I(a^{'},t) + \alpha I_b(a^{'},t)].$$

$I(a^{'},t)$ represents the number of infectious individuals (wild-type CP) in age class $a^{'}$ at time $t$. $I_b(a^{'},t)$ represents the number of infectious breakthrough CP cases at time $t$. $\beta$ is the age-independent proportionality factor and $\alpha$ is a parameter for reduced infectiousness of breakthrough CP cases in comparison with the wild-type. This value is assumed to be 0.5 [6]. HZ is assumed not to contribute in the transmission of CP in our model. $C(a,a^{'})$ is a contact matrix representing the per capita rates at which an individual of age class $a$ makes contacts with an individual of age class $a^{'}$, per unit time. The social contact matrix is based on social contact survey in Flanders in Belgium. For more details on the social contact data, see the data description Subsection 4. The force of boosting (FOB) results from multiplying the FOI with boosting factor $z\lambda(a,t)$ where $z$ represents the probability of boosting.

## 1.4 Model calibration

We employ the model component (Supplementary Figure 1**a**) representing the natural history of CP and HZ in absence of CP and HZ vaccination programs for the model calibration. We use data on CP sero-prevalence and HZ incidence data.

### 1.4.1 Fitting the CP sero-prevalence data

We take a two-step approach in the model fitting. Firstly, we estimate the proportionality parameter ($\beta$) and consequently the FOI given the CP sero-prevalence. Then we estimate the reactivation parameters of HZ ($z$, $q$, $\rho_0$, $\theta_a$, $\theta_\tau$) using the estimated ($\beta$). The proportionality factor $\beta$ represents the probability of getting infected given a contact between an infected and a susceptible individual and is approximated using the maximum likelihood approach by assuming the population is at endemic equilibrium. We use the MSEIR sub-component of the model involving the CP transmission component only as we assume HZ does not contribute to the force of infection (FOI).
Using this sub-component of the model, the population is discretised into 100 age groups. From the model, the probability that an individual of age $a$ is infected and thus sero-positive is equivalent to the age-specific sero-prevalence that can be expressed as:

$$\pi(a) = 1 - S(a)$$

where $S(a)$ represents the age-specific proportion of susceptible at endemic equilibrium.
If we denote $1_i$ the indicator variable for seropositivity ($1_i$=1 if sample $i$ is seropositive and $1_i$=0 if sample $i$ seronegative), and $a_i$ the age of the individual corresponding to sample $i$, then the Bernoulli log-likelihood of the CP serological data is computed as follows;

$$\ell_{VZV}(\beta|d_{vzv}) = \sum_{i=1}^{n}(1_i \log(\pi(a_i)) + (1-1_i)\log(1-\pi(a_i))$$

where $n$ is the number of samples in the serological data and $d_{vzv}$ is the CP sero-prevalence data. For each value of $\beta$, the MSEIR model is run till equilibrium to yield the proportions of the susceptible S(a), and subsequently $\pi(a)$.
The $\pi(a)$ which represents the age-specific sero-prevalence from the model is then contrasted to the age-specific sero-prevalence data up to 66 years. This results to a maximum likelihood estimate of $\beta$. Optimization is performed using the optim function in R using the Brent method. The profile likelihood method is used to compute the 95% confidence interval for $\beta$. Comparison of the CP sero-prevalence and the model predicted sero-prevalence is predicted in Supplementary Figure 2.

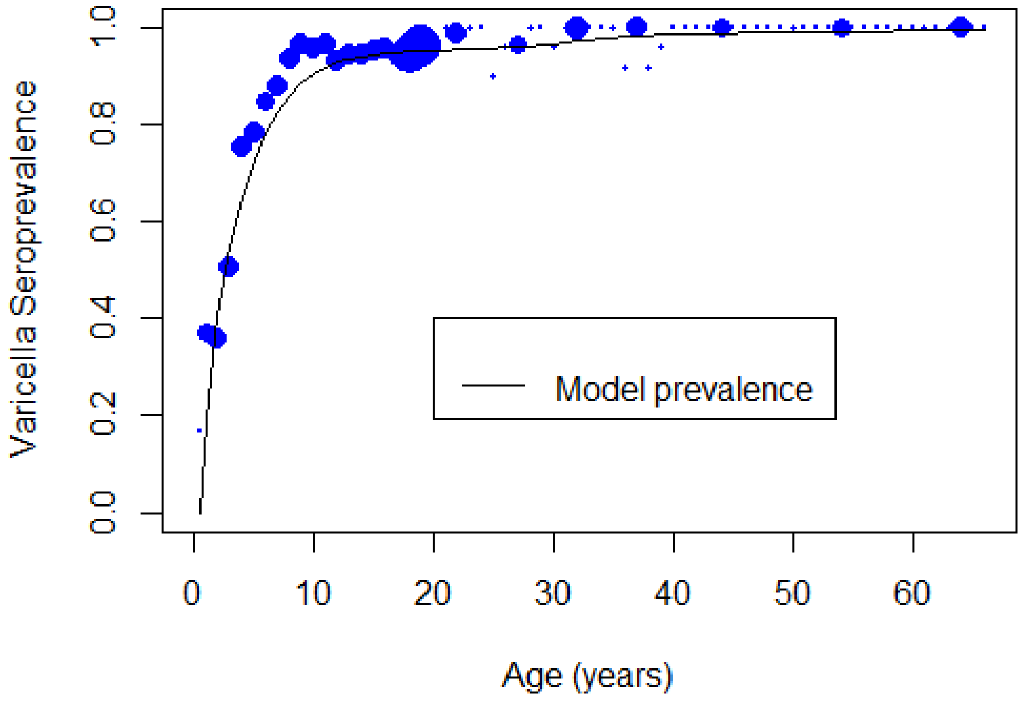


Supplementary Fig 2: CP seroprevalence (blue dots) and predicted sero-prevalence computed from the *Prog* model by the UA team (black line). The size of the dots reflect the sample sizes. The total number of sera is 3250.

#### 1.4.2 Fitting the HZ incidence data

To fit the age-specific HZ incidence data in order to estimate the reactivation related parameters ($z$,$q$,$\theta_a$,$\theta_\tau$,$\rho_0$), we employed a likelihood based approach by assuming;

$$Y_{k,j} = Poisson(I_{HZnew}(j) - I_{HZnew}(j-1)),$$

where $y_{k,j}$ is the new number of observed HZ cases in age group k, ($k = 1, 2, 3, ..., 100$) in year $j$ at endemic equilibrium. $I_{HZ}$ is the expected cumulative new number of HZ cases in age group $k$ obtained from numerically solving the dynamical model at endemic equilibrium. We applied maximum likelihood approach using the optim function in R and using the method "L-BFGS-B" - a limited memory algorithm for bound constrained optimization for solving large nonlinear optimization problems by Byrd et al. [8]. Comparison of the observed and predicted HZ incidence is depicted in Supplementary Figure 3.

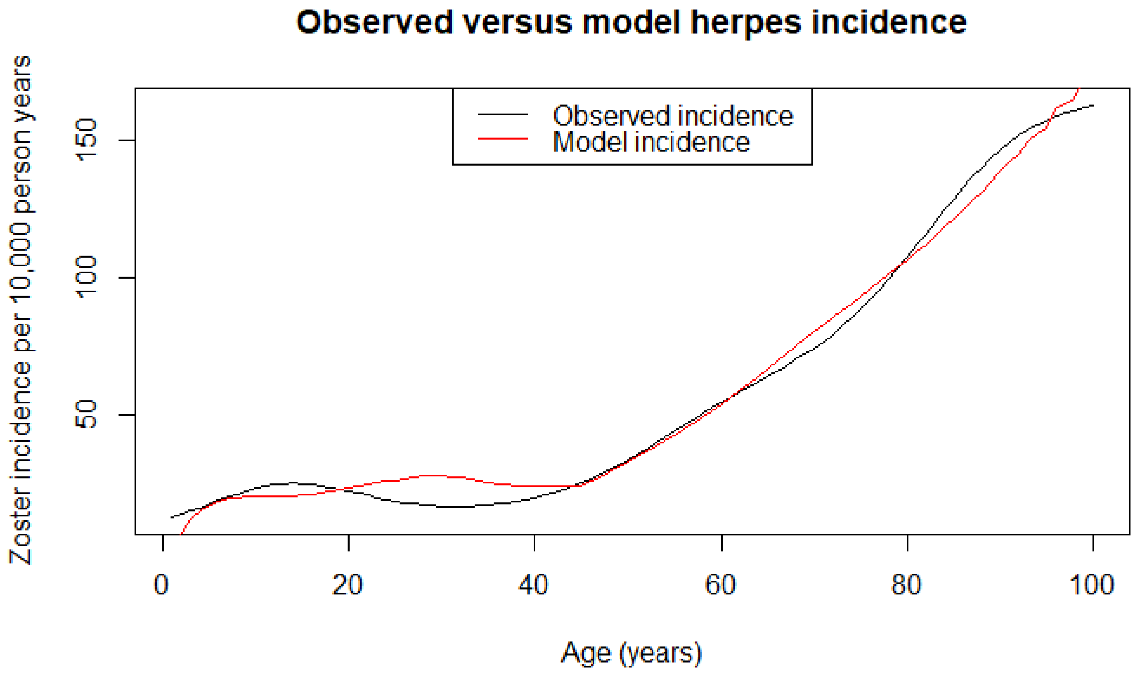


Supplementary Fig 3: Comparison of the observed HZ incidence and the predicted HZ incidence in the *Prog* model by the UA team.

| Parameter | Description of the parameter | Value | Source |
|---|---|---|---|
| $a_1$ | Age at routine vaccination against CP with the first dose (depends on the vaccination strategy) | 1 or 2 years | Authors' assumption |
| $a_2$ | Age at routine vaccination against CP with the second dose (depends on the vaccination strategy) | 5.5 or 8 years | Authors' assumption |
| $a_h$ | Age at routine vaccination against HZ | 60 years | Authors' assumption |
| $cv_1$ | CP vaccination coverage with first dose | 95% | Authors' assumption |
| $cv_2$ | CP vaccination coverage with second dose | 90% | Authors' assumption |
| $e_1$ | CP vaccine efficacy after the first dose (MSD) | 93.1% | [9] |
| $e_2$ | CP vaccine efficacy after the second dose (MSD) | Permanent | Authors' assumption |
| $m$ | Duration of passive immunity | 0.5 years | [10] |
| $1 / \omega_1$ | Average duration of waning of the first dose protection | 31 years | Computed |
| $z$ | Probability of boosting | 0.7107 | Calibration |
| $\chi$ | Attenuation coefficient of reduced susceptibility of reactivation | 10% | [7] |
| $cv_h$ | Vaccination coverage of the HZ | 50% | Authors assumption |
| $e_h$ | Vaccine efficacy of the HZ vaccine (Shingrix) | 91.3% | [11] |
| $\beta$ | Proportionality factor | 0.045 | Calibration |
| $1/\omega_h$ | Average waning period of HZ vaccine (Shingrix) | 49.0 years | Computed from [12] |
| $\rho_0$ | Risk of developing HZ for individuals who have recovered from CP | 0.002379 | Calibration |
| q | Parameter shaping the reduction of HZ risk due to re-exposure to VZV | 0.8202 | Calibration |
| $\theta_a$ | Parameter shaping the increased risk of HZ with age | 0.0524 | Calibration |
| $\theta_\tau$ | Parameter shaping the increased risk of HZ due to time since last exposure to VZV | 0.03803 | Calibration |
| $1/\gamma$ | Infectious period of wild-type CP | 7 days | [13, 14] |
| $1/\sigma$ | Latent period of wild-type CP/breakthrough CP | 14 days | [13, 14] |
| $1/\gamma_b$ | Infectious period of breakthrough CP | 4.5 days | [13, 15] |
| $1/\gamma_{hz}$ | Recovery period of HZ | 28 days | [13, 15] |

Supplementary Table 2: Epidemiological baseline parameter values for the CP and HZ vaccination programs in the UA compartmental model employing the progressive immunity boosting mechanism. We report the values and their source: whether taken from the literature, computed from available data or calibrated from the model.

Supplementary Table 3: Characteristics and source of input parameters used in the health economic model evaluation.

| Parameter | Estimate mean (95% Confidence interval) | | Uncertainty distribution | Source |
|---|---|---|---|---|
| Proportion of hospitalized CP patients not visiting a GP | 0.18 | | Beta ($\alpha$=10,$\beta$=47) | NCSF [16, 17] |
| Proportion of CP patients not visiting a GP | 0.79 | | Beta ($\alpha$=41,$\beta$=11) | NCSF [16, 17] |
| Proportion of ambulatory CP patients visiting only a specialist (no GP) | 0.29 | | Beta ($\alpha$=15,$\beta$=37) | NCSF [16, 17] |
| QALY loss ambulatory CP patient | 0.0054 (0.0053 - 0.0061) | | 1 - [N($\mu$=0.7182,$\sigma$=0.0106)/52] | [18] |
| QALY loss hospitalized CP patient | 0.0268 (0.0258 - 0.0290) | | 1 - [N($\mu$=0.1101,$\sigma$=0.0271)/52] | [18] |
| QALY loss CP patient not seeking medical care | 0.004 | | 1 - [N($\mu$=0.78,$\sigma$=0.04)/52] | NCSF [16, 17] |
| Cost of hospitalized CP patient | 3996.05 | | N ($\mu$= 3996.05,$\sigma$= 234.57) | NCSF [16, 17] |
| Cost of ambulatory CP patient | 49.97 | | N ($\mu$= 49.97,$\sigma$= 4.275) | NCSF [16, 17] |
| Case mortality rate for CP | | | | |
| Age group | | | | |
| 0-15 years | 0.0000005 | | | [16] |
| 16-100 years | 0.00000012 | | | [16] |
| Duration of HZ (days) for $<$ 70 years old | 28.66 (24.47 - 32.99) | | N ($\mu$= 28.66,$\sigma$= 2.170) | NCSF [16, 19] |
| Duration of HZ (days) for $\geq$ 70 years old | 44.38 (31.46 - 57.44) | | N ($\mu$= 44.38,$\sigma$= 3.785) | NCSF [16, 19] |
| Duration of PHN (days) for $<$ 70 years old | 254.06 (153.58 - 353.47) | | N ($\mu$= 254.06,$\sigma$= 50.74) | NCSF [16, 19] |
| Duration of PHN (days) for $\geq$ 70 years old | 319.46 (255.13 - 382.58) | | N ($\mu$= 319.46,$\sigma$= 33.20) | NCSF [16, 19] |
| Proportion of HZ and PHN according to pain severity | | | | |
| $<$ 70 years | HZ | PHN | | |
| No pain | 0.65 | NA | | [19, 20] |
| Mild pain | 0.24 | NA | | [19, 20] |
| Moderate pain | 0.04 | 0.89 | | [19, 20] |
| Severe pain | 0.08 | 0.11 | | [19, 20] |
| >= 70 years | HZ | PHN | | |
| No pain | 0.45 | NA | | [19, 20] |
| Mild pain | 0.41 | NA | | [19, 20] |
| Moderate pain | 0.05 | 0.89 | | [19, 20] |
| Severe pain | 0.09 | 0.11 | | [19, 20] |
| Case mortality rate for HZ and PHN | | | | |
| Age group | | | | |
| 0-59 | 0.0 | | | [19] |
| 60-74 | 0.00000018 | | | [19] |
| 75-89 | 0.0000054 | | | [19] |
| 90 plus | 0.00004 | | | [19] |
| QALY weight for HZ and PHN | | | | |
| QALY weight no pain | 0.8559 (0.7750 - 0.9212) | | Beta ($\alpha$=71.536$\beta$=12.132) | [19, 21] |
| QALY weight mild pain | 0.7787 (0.7509 - 0.8567) | | Beta ($\alpha$=78.621,$\beta$=7.775) | [19, 21] |
| QALY weight moderate pain | 0.6075 (0.5826 - 0.6788) | | Beta ($\alpha$=155.289,$\beta$=63.428) | [19, 21] |
| QALY weight severe pain | 0.2731 (0.2288 - 0.4030) | | Beta ($\alpha$=13.327,$\beta$=28.321) | [19, 21] |
| **Cost of ambulatory HZ patients** | | | | |
| Cost of ambulatory HZ patients with no pain | 30 | | | NCSF [19] |
| Cost of ambulatory HZ patients with mild pain | 101.85 (61.94 - 149.87) | | Gamma ($\alpha$=20.616,$\beta$=0.202) | NCSF [16, 19] |
| Cost of ambulatory HZ patients with moderate pain | 243.23 (168.93 - 330.45) | | Gamma ($\alpha$=34.846,$\beta$=0.143) | NCSF [16, 19] |
| Cost of ambulatory HZ patients with severe pain | 247.16 (137.35 - 387.28) | | Gamma ($\alpha$=15.028,$\beta$=0.061) | NCSF [16, 19] |
| **Cost of ambulatory PHN patients** | | | | |
| Cost of ambulatory PHN patients with moderate pain | 543.04 (330.17 - 796.98) | | Gamma ($\alpha$=20.794,$\beta$=0.0383) | NCSF [16, 19] |
| Cost of ambulatory PHN patients with severe pain | 842.79 (396.64 - 1377.75) | | Gamma ($\alpha$=11.339,$\beta$=0.0134) | NCSF [16, 19] |
| **Cost of hospitalized HZ patients** | | | | |
| Cost of hospitalized HZ patients with no pain | 6326.93 (4541.61 - 9153.77) | | Gamma ($\alpha$=28.916,$\beta$=0.0046) | NCSF [16, 19] |
| Cost of hospitalized HZ patients with mild pain | 6326.93 (4541.61 - 9153.77) | | Gamma ($\alpha$=28.916,$\beta$=0.0046) | NCSF [16, 19] |
| Cost of hospitalized HZ patients with moderate pain | 6600.254 (5663.77 - 7593.31) | | Gamma ($\alpha$=179.798,$\beta$=0.0272) | NCSF [16, 19] |
| Cost of hospitalized HZ patients with severe pain | 7301.86 (5219.26 - 9786.36) | | Gamma ($\alpha$=39.278,$\beta$=0.0054) | NCSF [16, 19] |
| **Cost of hospitalized PHN patients** | | | | |
| Cost of hospitalized PHN patients with moderate pain | 7408.66 (6151.68 - 8756.14) | | Gamma ($\alpha$=124.3415,$\beta$=0.0168) | NCSF [16, 19] |
| Cost of hospitalized PHN patients with severe pain | 12358.32 (8870.17 - 16444.22) | | Gamma ($\alpha$=40.9104,$\beta$=0.0033) | NCSF [16, 19] |

*NCSF* : National Christian Sickness Fund. *N* : denotes normal distribution, *Beta* : denotes beta distribution, *Gamma* : denotes gamma distribution

## 2 MSD compartmental model

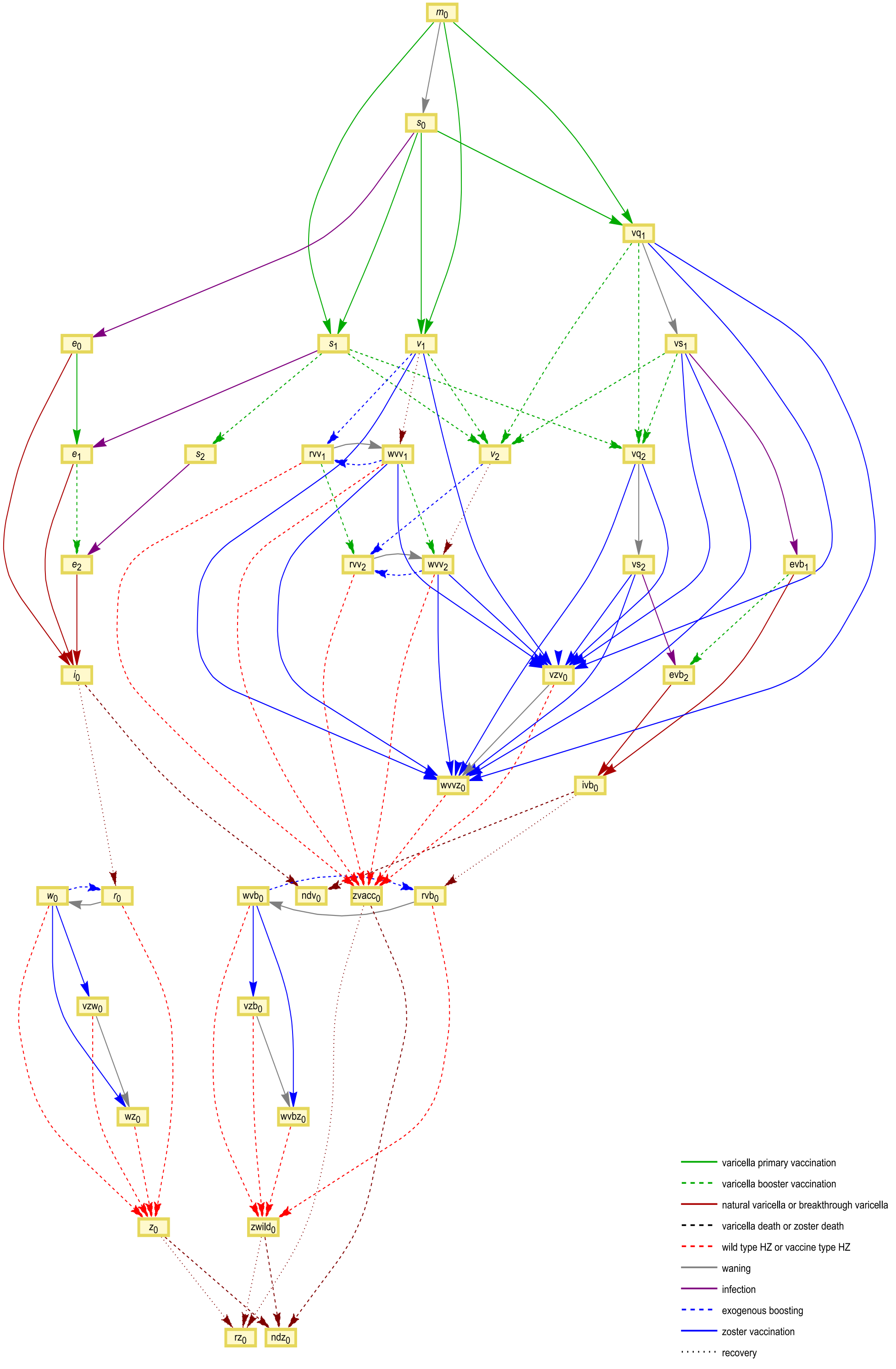


Supplementary Fig 4: Representation of the compartmental model structure with 53 age groups applied by MSD team for CP and HZ natural history and vaccine effects.

## 2.1 MSD model structure for CP and HZ

Newborns enter the model with passive immunity due to maternal antibodies ($m$). They become susceptible to natural CP ($s$) following waning of immunity from maternal antibodies after an average duration of 6 months. Without vaccination, children can be exposed to VZV ($e$) when they come into contact with individuals infected with CP or HZ. After an average duration of 14 days, exposed individuals become infectious ($i$). These infectious individuals then either recover from CP infection with high immunity against HZ ($r$) or die from CP ($ndv$). Over time, recovered individuals will wane acquired HZ immunity ($w$). Children are offered the first dose of CP at age ($a_1$). A vaccine dose may fail to induce an immune response, succeed in inducing temporary immunity ($vq$), or succeed in inducing long-lasting immunity ($v$). In the case of vaccine failure, children remain susceptible ($s$) to CP infection with outcomes that are severe as the wild-type CP. Immunity conferred from a successful first dose is either temporary ($vq_1$) or long-lasting ($v_1$). Temporary immunity wanes at a rate based on vaccine characteristics, and after waning, these individuals are at a risk of acquiring breakthrough CP. If individuals with waned vaccine immunity are exposed to VZV ($evb_1$), they will progress to infectious breakthrough CP ($ivb$). Breakthrough infectious individuals can either recover with high immunity against HZ ($rvb$) or die from CP ($ndv$). Individuals recovered from CP, can lose HZ acquired immunity ($wvb$).

For children who have received their first CP vaccine dose in a two-dose vaccine schedule, the second dose is administered at age ($a_2$). If the second dose is successful, immunity gained is either temporary ($vq_1$) or long-lasting ($v_{20}$). The rest of the dynamics follow the same trajectory as that after the first successful dose. As immunity against HZ wanes, individuals previously infected with VZV can undergo HZ reactivation ($z, zwild$). Individuals who have been previously and succesfully vaccinated against CP can undergo HZ reactivation as well from the vaccine-strain reactivation ($zvacc$). HZ reactivation results in recovery with life-long immunity ($rz$) or death ($ndz$). Throughout their lifetime, individuals can benefit from exogenous boosting. As described in the main text, VZV reactivation to HZ was modeled using the temporal immunity boosting assumption. Specifically, the model assumed that the likelihood of an exposure to CP infectious individual resulting in exogenous boosting was 33.45% and the average duration of full protection from the exogenous boosting was 81.3 years [22]. Individuals susceptible to VZV reactivation to HZ were vaccinated with the Shingrix vaccine from GSK. The Shingrix vaccine was assumed to confer temporary full immunity with an exponential waning rate ($\omega z$).

| Compartment | Description |
|---|---|
| Unvaccinated compartments | |
| $m_j$ | Passive immune |
| $s_j$ | susceptible to CP infection |
| $e_j$ | latent CP infection |
| $i_j$ | infectious CP infection |
| $r_j$ | recovered from CP with high HZ immunity |
| $w_j$ | low HZ immunity due to waning effects |
| CP vaccination | |
| $v_{j,l}$ | long-lasting immunity following $l-$ dose vaccination |
| $vq_{j,l}$ | temporary immunity following $l-$ dose vaccination |
| $vs_{j,l}$ | susceptible to CP following $l-$ dose vaccine waning |
| $s_{j,l}$ | susceptible to CP following $l-$ dose vaccine failure |
| $e_{j,l}$ | latent CP following $l-$ dose vaccine failure |
| $rvv_{j,l}$ | high HZ immunity following $l-$ dose vaccination |
| $wvv_{j,l}$ | low HZ immunity following $l-$ dose vaccination |
| Breakthrough CP | |
| $evb_{j,l}$ | latent CP following $l-$ dose vaccine waning |
| $ivb_j$ | infectious CP |
| $rvb_j$ | recovered from CP with high HZ immunity |
| $wvb_j$ | low HZ immunity due to waning |
| HZ reactivation | |
| $z_j$ | infectious with wild-type HZ |
| $zvacc_j$ | infectious with wild-type HZ post CP vaccination |
| $zwild_j$ | infectious with wild-type HZ post breakthrough CP |
| $rz_j$ | recovered from HZ with high HZ immunity |
| Death | |
| $ndv_j$ | death from CP |
| $ndz_j$ | death from HZ |

Supplementary Table 4: Description of the compartments in the MSD dynamical model.

## 3 Ordinary differential equations of the MSD model

$$\frac{dm_j}{dt} = B^m(t)\delta_{1,j} + d_{j-1}(t)(1-\delta_{1,j})(1-\theta_j^p(t)-\theta_j^c(t)-\theta_j^s(t))m_{j-1}(t) - (d_j(t)+\omega^m+\mu_j(t))m_j(t).$$

$$\frac{ds_j}{dt} = B^s(t)\delta_{1,j} + \omega^m m_j(t) + d_{j-1}(t)(1-\delta_{i,j})(1-\theta_j^p(t)-\theta_j^c(t)-\theta_j^s(t))s_{j-1}(t) - (d_j(t)+\mu_j(t)+\lambda_j(t))s_j(t).$$

$$\frac{de_j}{dt} = \lambda_j(t)s_j(t) + d_{j-1}(t)(1-\delta_{1,j})(1-\theta_j^p(t)-\theta_j^c(t)-\theta_j^s(t))e_{j-1}(t) - (d_j(t)+\mu_j(t)+\epsilon^n)e_j(t).$$

$$\frac{di_j}{dt} = d_{j-1}(t)(1-\delta_{1,j})i_{j-1}(t) + \epsilon^n(e_j(t)+e_{j,1}(t)+e_{j,2}(t)) - (d_j(t)+\mu_j(t)+\gamma^n+d_j^v)i_j(t).$$

$$\frac{dr_j}{dt} = d_{j-1}(t)(1-\delta_{1,j})r_{j-1}(t) + (\xi_j^n+\zeta_j^n\lambda_j)(t)w_j(t) + \gamma^n i_j(t) - (d_j(t)+\mu_j(t)+\delta^n+bxz\sigma_j)r_j(t).$$

$$\frac{dw_j}{dt} = d_{j-1}(t)(1-\delta_{1,j})(1-\theta_j^z(t))w_{j-1}(t) + \delta^n r_j(t) - (d_j(t)+\mu_j(t)+\sigma_j+\xi_j^n+\zeta_j^n\lambda_j(t))w_j(t).$$

$$\frac{dv_{j,1}}{dt} = d_{j-1}(t)(1-\delta_{1,j})v_{j-1,1}(t) + d_{j-1}(t)(1-\delta_{1,j})[(\theta_j^p(t)+\theta_j^c(t)+\theta_j^s(t))PT_1(m_{j-1}(t)+s_{j-1}(t)) -$$
$$(\theta_j^b(t)+\theta_j^d(t))v_{j-1,1}(t)] - (d_j(t)+\mu_j(t)+\pi_1+k_1\lambda_j(t))v_{j,1}(t).$$

$$\frac{dv_{j,2}}{dt} = d_{j-1}(t)(1-\delta_{1,j})v_{j-1,2}(t) + d_{j-1}(t)(1-\delta_{1,j})(\theta_j^b(t)+\theta_j^d(t))[v_{j-1,1}(t)+PT_1 s_{j-1,1}(t) +$$
$$T_2(vq_{j-1,1}(t)+vs_{j-1,1}(t))] - (d_j(t)+\mu_j(t)+\pi_2+k_2\lambda_j(t))v_{j,2}(t).$$

$$\frac{dvq_{j,1}}{dt} = d_{j-1}(t)(1-\delta_{1,j})vq_{j-1,1}(t) + d_{j-1}(t)(1-\delta_{1,j})[P(1-T_1)(\theta_j^p(t)+\theta_j^c(t)+\theta_j^s(t))(m_{j-1}(t)+s_{j-1}(t)) -$$
$$(\theta_j^b(t)+\theta_j^d(t)+\theta_j^z(t))vq_{j-1,1}(t)] - (d_j(t)+\mu_j(t)+\sigma^v)vq_{j,1}(t).$$

$$\frac{dvq_{j,2}}{dt} = d_{j-1}(t)(1-\delta_{1,j})(1-\theta_j^z(t))vq_{j-1,2}(t) + d_{j-1}(t)(1-\delta_{1,j})(\theta_j^b(t)+\theta_j^d(t))[P(1-T_1)s_{j-1,1}(t) +$$
$$(1-T_2)(vq_{j-1,1}(t)+vs_{j-1,1}(t))] - (d_j(t)+\mu_j(t)+\sigma^v)vq_{j,2}(t).$$

$$\frac{dvs_{j,1}}{dt} = \sigma^v vq_{j,1}(t) + d_{j-1}(t)(1-\delta_{1,j})(1-\theta_j^b(t)-\theta_j^d(t)-\theta_j^z(t))vs_{j-1,1}(t) - (d_j(t)+\mu_j(t)+\lambda_j(t))vs_{j,1}(t).$$

$$\frac{dvs_{j,2}}{dt} = \sigma^v vq_{j,2}(t) + d_{j-1}(t)(1-\delta_{1,j})(1-\theta_j^z(t))vs_{j-1,2}(t) - (d_j(t)+\mu_j(t)+\lambda_j(t))vs_{j,2}(t).$$

$$\frac{dvzv_j}{dt} = d_{j-1}(t)(1-\delta_{1,j})vzv_{j-1}(t) - T_z d_{j-1}(t)(1-\delta_{1,j})\theta_j^z(t)(vq_{j-1,1}(t)+vq_{j-1,2}(t)+vs_{j-1,1}(t)+vs_{j-1,2}(t) +$$
$$wvv_{j-1,1}(t)+wvv_{j-1,2}(t)) - (wz+d_j(t)+\mu_j(t)+bz\chi\sigma_j(t))vzv_j(t).$$

$$\frac{dvzw_j}{dt} = d_{j-1}(t)(1-\delta_{1,j})(vzw_{j-1}(t)+T_z\theta_j^z(t)w_{j-1}(t)) - (w^z+d_j(t)+\mu_j(t)+bz\sigma_j(t))vzw_j(t).$$

$$\frac{dvzb_j}{dt} = d_{j-1}(t)(1-\delta_{1,j})(vzb_{j-1}(t)+T_z\theta_j^z wvb_{j-1}(t)) - (w^z+d_j(t)+\mu_j(t)+bz\chi\sigma_j(t))vzb_j(t).$$

$$\frac{ds_{j,1}}{dt} = d_{j-1}(t)(1-\delta_{1,j})s_{j-1,1}(t) + d_{j-1}(t)(1-\delta_{1,j})[(1-P)(\theta_j^p(t)+\theta_j^c(t)+\theta_j^s(t))(m_{j-1}(t)+s_{j-1}(t)) -$$
$$(\theta_j^b(t)+\theta_j^d(t))s_{j-1,1}(t)] - (d_j(t)+\mu_j(t)+\lambda_j(t))s_{j,1}(t).$$

$$\frac{ds_{j,2}}{dt} = d_{j-1}(t)(1-\delta_{1,j})s_{j-1,2}(t) + d_{j-1}(t)(1-\delta_{1,j})(1-P)(\theta_j^b(t)+\theta_j^d(t))s_{j-1,1}(t) -$$
$$(d_j(t)+\mu_j(t)+\lambda_j(t))s_{j,2}(t).$$

$$\frac{de_{j,1}}{dt} = d_{j-1}(t)(1-\delta_{1,j})e_{j-1,1}(t) + d_{j-1}(t)(1-\delta_{1,j})[(\theta_j^p(t)+\theta_j^c(t)+\theta_j^s(t))e_{j-1}(t) - (\theta_j^b(t)+\theta_j^d(t))e_{j-1,1}(t)] +$$
$$\lambda_j(t)s_{j,1}(t) - (d_j(t)+\mu_j(t)+\epsilon^n)e_{j-1}(t).$$

$$\frac{de_{j,2}}{dt} = d_{j-1}(t)(1-\delta_{1,j})e_{j-1,2}(t) + d_{j-1}(t)(1-\delta_{1,j})(\theta_j^b(t)+\theta_j^d(t))e_{j-1,1}(t) + \lambda_j(t)s_{j,2}(t) - (d_j(t)+\mu_j(t)+\epsilon^n)e_{j,2}(t).$$

$$\frac{drvv_{j,1}}{dt} = d_{j-1}(t)(1-\delta_{1,j})(1-\theta_j^b(t)-\theta_j^d(t))rvv_{j-1,1}(t) + \xi_j^{vv}wvv_{j,1}(t) + \lambda_j(t)[k_1v_{j,1}(t)+\zeta_j^{vv}wvv_{j,1}(t)] - (d_j(t)+\mu_j(t)+bxz\chi\sigma_j+\delta^{vv})rvv_{j,1}(t).$$

$$\frac{drvv_{j,2}}{dt} = d_{j-1}(t)(1-\delta_{1,j})[(\theta_j^b(t)+\theta_j^d(t))rvv_{j-1,1}(t)+rvv_{j-1,2}(t)] + \xi_j^{vv}wvv_{j,2}(t) + \lambda_j(t)[k_2v_{j,2}(t) + \zeta_j^{vv}wvv_{j,2}(t)] - (d_j(t)+\mu_j(t)+bxz\chi\sigma_j+\delta^{vv})rvv_{j,2}(t).$$

$$\frac{dwvv_{j,1}}{dt} = d_{j-1}(t)(1-\delta_{1,j})(1-\theta_j^b(t)-\theta_j^d(t)-\theta_j^z(t))wvv_{j-1,1}(t) + \delta^{vv}rvv_{j,1}(t) + \pi_1v_{j,1}(t) - (d_j(t)+\mu_j(t) + X\sigma_j+\xi_j^{vv}+\zeta_j^{vv}\lambda_j(t))wvv_{j,1}(t).$$

$$\frac{dwvv_{j,2}}{dt} = d_{j-1}(t)(1-\delta_{1,j})(1-\theta_j^z(t))wvv_{j-1,2}(t) + d_{j-1}(t)(1-\delta_{1,j}))(\theta_j^b(t)+\theta_j^d(t))wvv_{j-1,1}(t) + \delta^{vv}rvv_{j,2}(t) + \pi_2v_{j,2}(t) - (d_j(t)+\mu_j(t)+\chi\sigma_j+\xi_j^{vv}+\zeta_j^{vv}\lambda_j(t))wvv_{j,2}(t).$$

$$\frac{dwz_j}{dt} = \omega^z vzw_j(t) + d_{j-1}(t)(1-\delta_{1,j})[(1-T_z)\theta_j^z(t)w_{j-1}(t)+wz_{j-1}(t)] - (d_j(t)+\mu_j(t)+\sigma_j)wz_j(t).$$

$$\frac{devb_{j,1}}{dt} = d_{j-1}(t)(1-\delta_{1,j})(1-\theta_j^b(t)-\theta_j^d(t))evb_{j-1,1}(t) + \lambda_j(t)vs_{j,1}(t) - (d_j(t)+\mu_j(t)+\epsilon^{vb})evb_{j,1}(t).$$

$$\frac{devb_{j,2}}{dt} = d_{j-1}(t)(1-\delta_{1,j})evb_{j-1,2}(t) + \lambda_j(t)vs_{j,2}(t) + d_{j-1}(t)(1-\delta_{1,j}(t))(\theta_j^b(t)+\theta_j^d(t)evb_{j-1,1}(t) - (d_j(t)+\mu_j(t)+\epsilon^{vb})evb_{j,2}(t).$$

$$\frac{divb_j}{dt} = d_{j-1}(t)(1-\delta_{1,j})ivb_{j-1}(t) + \epsilon^{vb}(evb_{j,1}(t)+evb_{j,2}(t)) - (d_j(t)+\mu_j(t)+\gamma^{vb}+d_j^{vb})ivb_j(t).$$

$$\frac{drvb_j}{dt} = d_{j-1}(t)(1-\delta_{1,j})rvb_{j-1}(t) + (\xi_j^{vb}+\zeta^{vb}\lambda_j(t))wvb_j(t) + r^{vb}ivb_j(t) - (d_j(t)+\mu_j(t)+\delta^{vb}+bxz\chi\sigma_j)rvb_j(t)$$

$$\frac{dwvb_j}{dt} = d_{j-1}(t)(1-\delta_{1,j})(1-\theta_j^z(t))wvb_{j-1}(t) + \delta^{vb}rvb_j(t) - (d_j(t)+\mu_j(t)+\chi\sigma_j+\xi_j^{vb}+\zeta_j^{vb}\lambda_j(t))wvb_j(t).$$

$$\frac{dwvbz_j}{dt} = \omega^z vzb_j(t) + d_{j-1}(t)(1-\delta_{1,j})[(1-T_z)\theta_j^z(t)wvb_{j-1}(t)+wvbz_{j-1}(t)] - (d_j(t)+\mu_j(t)+\sigma_j)wvbz_j(t).$$

$$\frac{dwvvz_j}{dt} = \omega^z vzv_j(t) + d_{j-1}(t)(1-\delta_{1,j})[(1-T_z)\theta_j^z(t)(vq_{j-1,1}(t)+vq_{j-1,2}(t)+vs_{j-1,1}(t)+vs_{j-1,2}(t) + wvv_{j-1,1}(t)+wvv_{j-1,2}(t)+wvvz_{j-1}(t))] - (d_j(t)+\mu_j(t)+\chi\sigma_j)wvvz_j(t).$$

$$\frac{dz_j}{dt} = \sigma_j(bxzr_j(t)+bzvzw_j(t)+w_j(t)+wz_j(t)) + d_{j-1}(t)(1-\delta_{1,j})z_{j-1}(t) - (d_j(t)+\mu_j(t)+\eta^n+d_j^z)z_j(t).$$

$$\frac{dzvacc_j}{dt} = d_{j-1}(t)(1-\delta_{1,j})zvacc_{j-1}(t) + \chi\sigma_j(bxzrvv_{j,1}(t)+bzxrvv_{j,2}(t)+bzvzv_j(t)+wvv_{j,1}(t)+wvv_{j,2}(t) + wvvz_j(t)) - (d_j(t)+\mu_j(t)+\eta^{vv}+d_j^z)zvacc_j(t).$$

$$\frac{dzwild_j}{dt} = d_{j-1}(t)(1-\delta_{1,j})zwild_{j-1}(t) + \chi\sigma_j(bxzrvvb_j(t)+bzvzb_j(t)+wvb_j(t)+wvbz_j(t)) + (d_j(t)+\mu_j(t)+\eta^{vb}+d_j^z)zwild_j(t).$$

$$\frac{drz_j}{dt} = d_{j-1}(t)(1-\delta_{1,j})rz_{j-1}(t) + \eta^n z_j(t) + \eta^{vb}zwild_j(t) + \eta^{vv}zvacc_j(t) - (d_j(t)+\mu_j(t))rz_j(t).$$

$$\frac{dndv_j}{dt} = d_{j-1}(t)(1-\delta_{1,j})ndv_{j-1}(t) + d_j^v i_j(t) + d_j^{vb} ivb_j(t) - (d_j(t) + \mu_j(t))ndv_j(t).$$
$$\frac{dndz_j}{dt} = d_{j-1}(t)(1-\delta_{1,j})ndz_{j-1}(t) + d_j^z(z_j(t) + zwild_j(t) + zvacc_j(t)) - (d_j(t) + \mu_j(t))ndz_j(t).$$

$$\lambda_j(t) = \sum_a \beta_{j,a}(i_a(t) + \rho^v ivb^a(t) + \rho^z z_a(t) + \rho^z zwild_a(t) + \rho^z zvacc_a(t)).$$

$$B^m(t) = \Lambda(t) - \frac{\Lambda(t)}{\sum_j f_j(t)n_j} \sum (f_j(t)s_j(t) + \sum f_j(t)s_{j,l}(t)).$$

$$B^s(t) = \frac{\Lambda(t)}{\sum_j f_j(t)n_j} \sum (f_j(t)s_j(t) + \sum f_j(t)s_{j,l}(t)).$$

$$\Lambda(t) = \cup(0,t) = \int_0^\infty f(a,t) \cup (a,t)\, dx, t \geq 0.$$

$$d_j(t) \equiv \frac{\cup(a_j,t)}{\int_{a_{j-1}}^{a_j} \cup(a,t)da}.$$

$$\cup(a,t) = \begin{cases} \cup(a-t,0)exp(-\int_0^t \bar{\omega}(a-t+\tau,\tau)d\tau), & \text{if } t \geq a, \\ \Lambda(t-a)(-\int_0^a \bar{\omega}(\tau, t-a+\tau)d\tau)), & \text{if } t \geq a. \end{cases}$$

No vaccination strategy:

$$\theta_j^p(t) = \theta_j^c(t) = \theta_j^b(t) = \theta_j^d(t) = 0$$

## 3.1 Model calibration

The model was calibrated to VZV sero-prevalence and HZ incidence data. The calibration process involved taking certain parameters governing the transmission of VZV infection and the natural history of HZ outbreaks, then finding maximum likelihood estimates of these parameters with respect to the epidemiological data.
The calibration proceeded in three steps:

1. Parameters were divided into those that primarily affect primary CP infection (e.g., relative risk parameters) and those that primarily affect HZ reactivation (e.g., HZ reactivation parameters).

2. HZ-related parameters are held constant and the CP-related parameters are optimized by minimizing the negative log likelihood function using a Nelder Mead algorithm [23] as implemented by the NMinimize routine in Mathematica.

3. The resulting maximum likelihood estimates of the CP parameters in step (2) are held constant and the HZ-related parameters are optimized by minimizing the negative log likelihood function using a Nelder Mead algorithm [23] as implemented by the NMinimize routine in Mathematica.

4. HZ parameters resulting from (3) are held constant and the CP-related parameters are optimized by minimizing the negative log likelihood function using a Nelder Mead algorithm [23] as implemented by the NMinimize routine in Mathematica.

The comparison plots for the VZV sero-prevalence in the MSD model are depicted in Supplementary Figure 5. Whilst the comparison plots for the observed and predicted HZ incidence are depicted in Supplementary Figure 6.

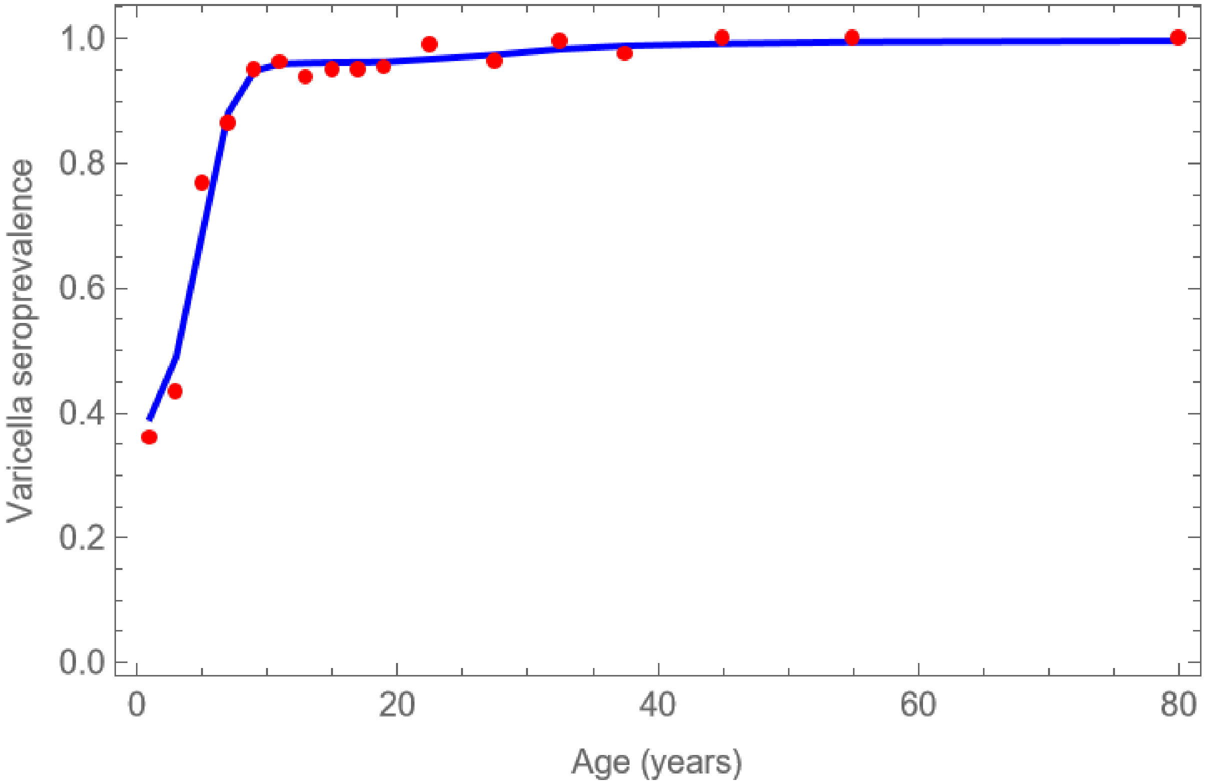


Supplementary Fig 5: Observed CP sero-prevalence (red dots) and predicted sero-prevalence computed from the *Temp* model by the MSD team (blue line).

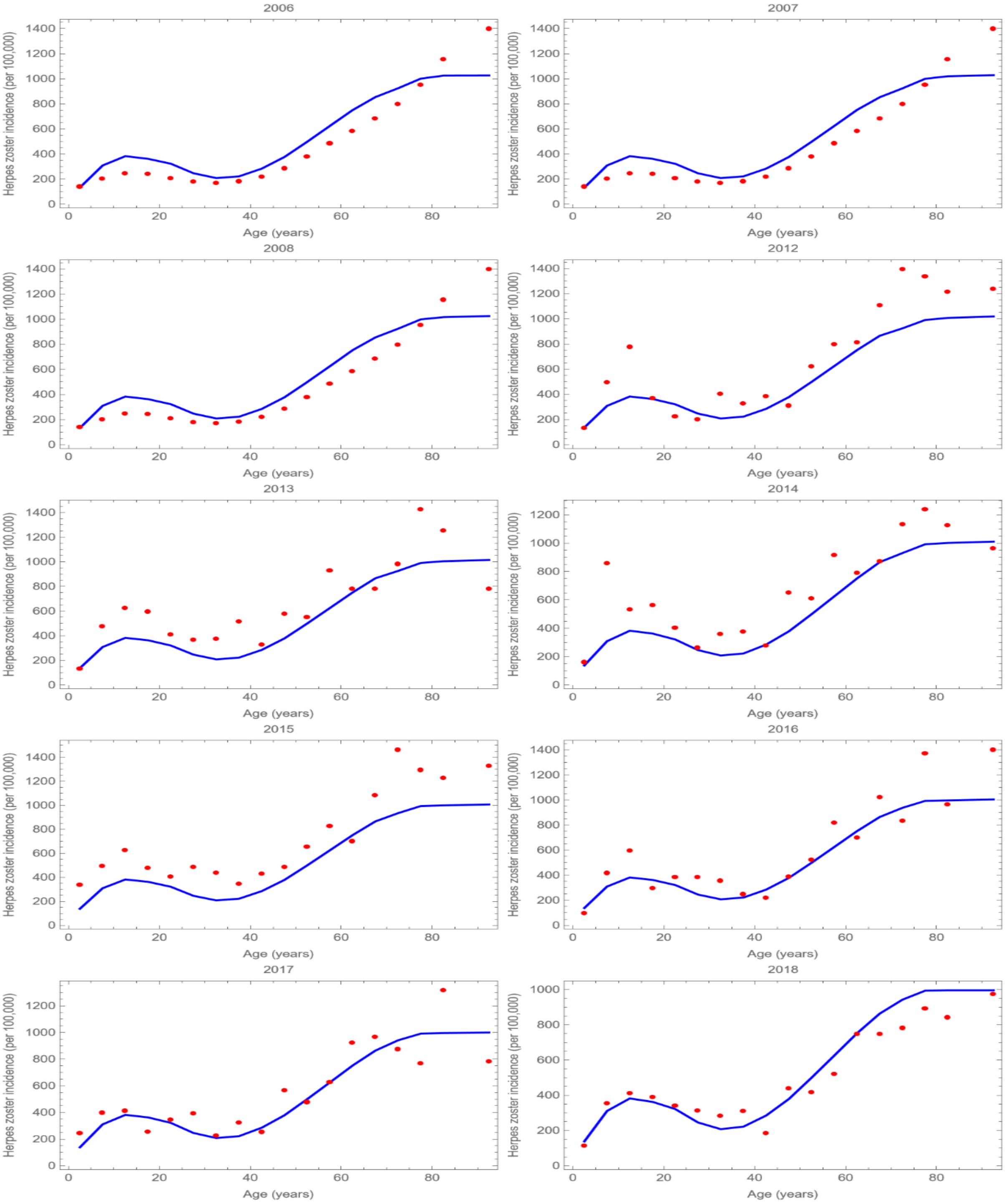


Supplementary Fig 6: Comparison of the observed HZ incidence and the predicted HZ incidence in the *Temp* model by MSD team.

| Parameter symbol | Description | Value | Source |
|---|---|---|---|
| | Natural CP infection | | |
| $1/\omega^m$ | Waning period of passive immunity | 6 months | [15, 10] |
| $1/\epsilon^n$ | Latent period of natural CP | 14 days | [10, 14] |
| $1/\gamma^n$ | Infectious period of natural CP | 7 days | [10, 13] |
| $1/\delta^n$ | Waning period of HZ immunity | 81.3 years | [22] |
| | Breakthrough CP infection | | |
| $1/\rho^v$ | Relative infectivity of breakthrough CP | 50% | [6] |
| $1/\epsilon^{vb}$ | Latent period of breakthrough CP | 14 days | [10, 14] |
| $1/\gamma^{vb}$ | Infectious period of breakthrough CP | 4.5 days | [10, 13] |
| $1/\delta^{vb}$ | Waning period after breakthrough CP of wild-type HZ | 81.3 years | [22] |
| | CP susceptibility | | |
| $rr_1$ | Relative risk (< 5 years old) | 0.0528 | Calibrated |
| $rr_2$ | Relative risk (5-10 years old) | 0.1323 | Calibrated |
| $rr_3$ | Relative risk (10-20 years old) | 0.0093 | Calibrated |
| $rr_4$ | Relative risk (≥20 years old) | 0.0580 | Calibrated |
| | HZ Reactivation | | |
| $\rho^z$ | Relative infectivity of HZ | 7% | |
| $\omega$ | HZ Reactivation parameter | 0.3365 | Calibrated |
| $\phi$ | HZ Reactivation parameter | 0.1171 | Calibrated |
| $\eta$ | HZ Reactivation parameter | 3.0686 | Calibrated |
| $\pi$ | HZ Reactivation parameter | 0.0142 | Calibrated |
| $\chi$ | Reactivation rate factor on vaccine arms | 1/6 | [24] |
| $1/\eta^n$ | Infectious period after natural CP of wild-type HZ | 28 days | [15, 25] |
| $1/\eta^{vb}$ | Duration of HZ outbreak following breakthrough CP | 28 days | [15] |
| $1/\eta^{vv}$ | Infectious period following successful vaccination of wild-type HZ | 28 days | [15] |
| $1/\delta^{vv}$ | Average waning period of exogenously boosted HZ immunity following successful vaccination | 81.3 years | [15] |
| | Contacts leading to exogenous boosting | | |
| $\zeta^n$ | after natural CP | 33.45% | [26] |
| $\zeta^{vb}$ | after breakthrough CP | 33.45% | [26] |
| $\zeta^{vv}$ | after CP vaccination | 33.45% | [26] |
| | Disease-related death, by year of age group | | |
| $d_j^v$ | CP infection-related | | |
| | 0-5 | 0.53 x $10^{-5}$ | [27] |
| | 5-10 | 0.28 x $10^{-5}$ | [27] |
| | 10-15 | 1.14 x $10^{-5}$ | [27] |
| | 15-20 | 0.86 x $10^{-5}$ | [27] |
| | 20-40 | 4.90 x $10^{-5}$ | [27] |
| | ≥ 40 | 3.190 x $10^{-4}$ | [27] |
| $d_j^z$ | Breakthrough CP infection-related | | |
| | 0-100 | 0.0000 | Assumption |
| $d_j^z$ | HZ reactivation-related death rate | | |
| | 0-60 | 0.0000 | [19] |
| | 70-75 | 1.79 x $10^{-5}$ | [19] |
| | 75-80 | 4.743 x $10^{-4}$ | [19] |
| | 80-90 | 4.955 x $10^{-4}$ | [19] |
| | ≥ 90 | 9.034 x $10^{-5}$ | [19] |

Supplementary Table 5: Epidemiological baseline parameter values for the CP and HZ vaccination programs in the MSD compartmental model employing the temporal immunity boosting mechanism. We report the values and their source: whether taken from the literature, computed from available data or calibrated from the model.

| Parameter symbol | Description | Value | Source |
|---|---|---|---|
| | CP treatment costs | | |
| Total direct CP treatment cost per CP infection | <5 | €32.73 | [16] |
| | 5-10 | €13.36 | [16] |
| | 10-15 | €48.29 | [16] |
| | 15-20 | €36.80 | [16] |
| | 20-60 | €77.05 | [16] |
| | 60-65 | €64.03 | [16] |
| | $\geq$ 65 | €64.03 | [16] |
| CP GP consultation cost | 0-100 | €44.25 | [16] |
| CP hospitalization cost | 0-100 | €3,538.29 | [16] |
| | HZ treatment costs | | |
| Direct HZ treatment cost per HZ episode | <5 | €260.81 | [16, 19] |
| | 5-10 | €115.63 | [16, 19] |
| | 10-40 | €137.98 | [16, 19] |
| | 40-50 | €165.66 | [16, 19] |
| | 50-60 | €230.36 | [16, 19] |
| | 60-65 | €293.60 | [16, 19] |
| | 65-70 | €293.60 | [16, 19] |
| | 70-80 | €408.16 | [16, 19] |
| | 80-90 | €635.77 | [16, 19] |
| | $\geq$90 | €470.89 | [16, 19] |
| | HZ GP consultation costs | | |
| | < 5 | €67.17 | [16, 19] |
| | 5-10 | €67.17 | [16, 19] |
| | 10-40 | €67.17 | [16, 19] |
| | 40-50 | €67.17 | [16, 19] |
| | 50-60 | €107.57 | [16, 19] |
| | 60-65 | €121.04 | [16, 19] |
| | 65-70 | €121.04 | [16, 19] |
| | 70-80 | €153.42 | [16, 19] |
| | 80-90 | €168.62 | [16, 19] |
| | $\geq$90 | €164.28 | [16, 19] |
| | HZ hospitalization costs | | |
| | < 5 | €5,767.22 | [16, 19] |
| | 5-10 | €5,767.22 | [16, 19] |
| | 10-40 | €5,767.22 | [16, 19] |
| | 40-50 | €5,767.22 | [16, 19] |
| | 50-60 | €5,889.52 | [16, 19] |
| | 60-65 | €5,930.28 | [16, 19] |
| | 65-70 | €5,930.28 | [16, 19] |
| | 70-80 | €5,996.58 | [16, 19] |
| | 80-90 | €6,043.93 | [16, 19] |
| | $\geq$ 90 | €6,030.40 | [16, 19] |

Supplementary Table 6: Health economic parameter values for the CP and HZ vaccination programs in the MSD compartmental model employing the temporal immunity boosting mechanism. We report the values and their source: whether taken from the literature, computed from available data or calibrated from the model.

| Symbol | Description | Mean | Standard deviation | Distribution |
|---|---|---|---|---|
| | | Natural Varicella | | |
| | Maternal immunity waning rate (per year) | 10% | 2% | Lognormal |
| | Fraction of contacts that lead to exogenous boosting | 0.3345 | 20% | Beta |
| | Waning rate for high HZ immunity state | 0.01230 | 20% | Lognormal |
| | Recovery rate for HZ (per year) | 13.04 | 20% | Lognormal |
| | | Breakthrough Varicella | | |
| | Waning rate for high HZ immunity state (following breakthrough VZV infection) (per year) | 0.01230 | 20% | Lognormal |
| | Fraction of contacts that lead to exogenous boosting | 0.3345 | 20% | Beta |
| | Recovery rate for HZ (per year) | 13.04 | 20% | Lognormal |
| | | Varicella Vaccination | | |
| | Waning rate for high HZ immunity state (following VZV vaccination) (per year) | 0.01230 | 20% | Lognormal |
| | Fraction of contacts that lead to exogenous boosting | 0.3345 | 20% | Beta |
| | Recovery rate for HZ (per year) | 13.04 | 20% | Lognormal |
| | Relative infectiousness of vaccinated individuals | 0.5 | 20% | Beta |
| | Relative reactivation rate for HZ (in VZV or HZ vaccinated individuals) | 0.1667 | 20% | Beta |
| | | Herpes Zoster | | |
| | Relative infectiousness of HZ | 0.07 | 20% | Beta |
| | Duration of postherpetic neuralgia (years) | 0.75 | 20% | Lognormal |
| | | Scaling Factors | | |
| | Scaling factor for direct VZV vaccination costs | 1 | 10% | Lognormal |
| | Scaling factor for direct VZV treatment costs | 1 | 10% | Lognormal |
| | Scaling factor for indirect VZV treatment costs | 1 | 10% | Lognormal |
| | Scaling factor for direct HZ vaccination costs | 1 | 10% | Lognormal |
| | Scaling factor for direct HZ treatment costs | 1 | 10% | Lognormal |
| | Scaling factor for indirect HZ treatment costs | 1 | 10% | Lognormal |
| | Scaling factor for natural varicella QALYs | 1 | 5% | Scaled Beta |
| | Scaling factor for breakthrough varicella QALYs | 1 | 5% | Scaled Beta |
| | Scaling factor for HZ QALYs (without PHN) | 1 | 5% | Scaled Beta |
| | Scaling factor for HZ QALYs (with PHN) | 1 | 5% | Scaled Beta |

Supplementary Table 7: Parameter distributions for probabilistic sensitivity analyses in the MSD model. To reduce the dimensionality of the sensitivity analyses, the effect of multiple parameters were aggregated together through the use of a scaling factor.

# 4 Dataset description

## 4.1 CP sero-prevalence

The age-specific seroprevalence profiles of CP were obtained from a seroprevalence survey performed on a representative national serum banks in Belgium, Finland, England and Wales, Poland and Italy [28]. The sera were collected in the period between 1995 and 2004 from residual sera submitted for routine testing in laboratories in the different countries. In Belgium, the sera were collected between 2001-2003. In total, we included 3250 samples of participants from Belgium who were aged 6 months or older in order to avoid interference of maternal antibodies.

## 4.2 HZ incidence

This data was computed from the age-specific number of people who visited a general practitioner (GP) at least once for HZ from a sentinel surveillance system consisting of 150 general practitioners coordinated by the Scientific Institute of Public Health (now Sciensano) covering 1.5% of the Belgian population [16]. The participating GPs are representative in terms of age and gender. The SIPH provided data comprising the age-specific number of individuals who visited a GP at least once for HZ in the years 2006 to 2008. See Bilcke et al. [16] for more details.

## 4.3 Social contact data

The social contact data utilized in this study was obtained from a social contact survey contacted in Flanders in Belgium between September 2010 and February 2011 [29]. In total, the survey included 1774 participants recruited through the means of random digit dialing on landlines and mobile phones, with quota sampling by sex, age and geographical location. A contact was defined as a two-way conversations where at least three words were exchanged or contacts that entailed skin-to-skin touching. Participants were requested to record their social contacts using a paper diary during a randomly assigned day. The contact rates utilized in this work encompassed the total number of contacts (i.e, both the physical and non-physical contacts).

## 4.4 Age-specific mortality rate predictions

The mortality rates data utilized in this study was obtained from STATBEL (Statistics Belgium) which makes a number of datasets freely available for both commercial as well as non-commercial purposes [2]. The data spanned from 1992-2070 in one year age groups. We assumed that the age-specific mortality rates for the years 2071 to 2123 would be the same as the data for the year 2070.

## 4.5 Age-specific population predictions

The age specific population estimates were obtained from STATBEL (Statistics Belgium) [2]. The data spanned from 1992-2071 in one year age groups.

## 4.6 Fertility rates

The age-specific fertility rates data for Belgium was obtained from the United Nations world population prospects. The data spanned the years 2022-2100 [3]. We assumed that the fertility rate data for the years 2101 to 2123 would be the same as the data for the year 2100.

## 4.7 Migration data

Age-specific migration schedules were modeled using the Rogers and Castro model implemented in the Demotools package in the R statistical software [4]. This mathematical model was developed in 1981 to model migration and comprises of 13 parameters [5].

## 4.8 Quality adjusted life years (QALYs) for wild type CP

The loss in quality of life due to CP disease infection was estimated from a survey conducted through the Child and Family Consultation services, an agency by the Flemish government

tasked with responsibility of families and young children in Flanders. It focuses on child care services, family support, preventive care and emotional support, diversity and children's rights.(https://cchp.nhs.uk/cchp/explore-cchp/child-family-consultation-services-camhs-0, assessed 21st July 2023). The survey was conducted in the period between February 2010 and July 2010. The quality of life lost for each CP patient was obtained as 1 minus EQ-5D-3L score and multiplying by the number of sick days. This was done separately for cases consulting a physician (General Practitioner (GP) as well as pediatricians) and those not consulting a physician [17]. From this data, the QALY loss of an hospitalized CP patient was 0.017 derived from a point estimate of one respondent [16, 17]. The QALY loss of CP patient not seeking medical care was 0.004 [16]. The QALY loss of an ambulatory CP patient was 0.010 [16]. We further obtained QALY estimates from a recent prospective study conducted in Portugal to assess the loss of health-related quality adjusted life years in children suffering from CP infection (uncomplicated and hospitalised). The study showed that for ambulatory treated CP patients, the QALY loss was 5.4 QALYs/1000 children (95 % CI 5.3–6.1) and 1.3 days/primary carer (95 % CI 1.2–1.6, n = 103) (mean 3.6 QALYs /1000 carers (95 % CI 3.4–4.4) [18]. For hospitalized VZV patients, the QALY loss was 26.8 QALYs/1000 children (95 % CI 25.8–29.0) and 23.4 QALYs/1000 carers (95 % CI 20.3–26.2). In our study, we utilize the QALY values for hospitalized and ambulatory treated CP patients from the Portugal study by Rodrigues et al [18]. Whilst the QALY values for CP cases not seeking medical care were based on the values from the Child and Family survey in Belgium [16]. More description on how these quality of life estimates were computed are described in more details in Rodrigues et al. [18] and Bilcke et al. [16].

### 4.9 CP and HZ general practitioner (GP) consultation rates

This data was computed from the age-specific number of people who visited a general practitioner (GP) at least once for CP and HZ from a sentinel surveillance system consisting of 150 general practitioners coordinated by the Scientific Institute of Public Health (now Sciensano) covering 1.5% of the Belgian population [16]. The participating GPs are representative in terms of age and gender. The SIPH provided data comprising the age-specific number of individuals who visited a GP at least once for CP or HZ in the years 2006 to 2008. See Bilcke et al. [16] for more details.

### 4.10 CP and HZ hospitalization rates

The age-specific rate of hospitalization for patients suffering from CP or HZ were obtained from the Minimal Clinical Data (MCD) by Bilcke et al. [16]. MCD is a mandatory registration for patients who are hospitalized in Belgium. The data spanned the period between 2000 to 2007. The annual rates of hospitalization for the CP and HZ were obtained by dividing the yearly number of CP and HZ hospitalizations from the MCD data with the population estimates in the same year.

### 4.11 Proportions of CP health care seeking

Since in Belgium it is possible to consult a specialist directly (e.g paediatrician or dermatologist) without having to consult a GP first [16], the proportions of hospitalized CP not visiting a GP were obtained from the National Christian Sickness Fund (NCSF) and Child and Family surveys. NCSF is a Belgian health insurance which comprised of 43.7% of the total population in Belgium. The NCSF contains medical information of its members. The NCSF survey was

based on the NCSF members. Further, the proportions of ambulatory CP patients not visiting a GP and proportions of ambulatory CP patients visiting only specialist (no GP) were obtained from the Child and Family surveys (Bilcke et al. [16, 17]). The proportion of hospitalized CP patients not visiting a physician in ambulatory care was 0.18 relative to the estimated number of people hospitalized because of CP. The proportions of CP patients not visiting a GP was 0.79 relative to those who consulted a GP at least once. This will apply for ages 0 to 12 years. For ages 13 and above, this proportion is assumed to be 0 similar to the assumption made by Bilcke et al. [17] where in view of the severity of the primary CP in teenagers and adults, it is assumed - rather than estimated that individuals aged more than 12 years will always seek medical care when they have a primary CP infection (either by consulting a physician or by being hospitalized). Whilst the proportion of ambulatory CP patients visiting only a specialist (no GP) is 0.29 relative to those who consulted a GP at least once. See Bilcke et al. [16] for more details on how these values were computed.

### 4.12 CP and HZ mortality rates

The mortality rates for CP and HZ were obtained from Bilcke et al. [16]. These rates were computed by acquiring all death certificates in the period 1998-2007 from the Flemish Agency for Care and Health for the residents of the flemish part of Belgium which represents approximately 60% of the Belgian population. The death certificates were screened by expert opinions to determine the ones related to CP and HZ. More information is given by Bilcke et al [16] and Pieters et al. [19].

### 4.13 Proportion of Post-herpetic Neuralgia (PHN)

Post-herpetic Neuralgia (PHN) is a painful condition often defined as pain persisting for more than 3 months after rash onset [30]. The age-specific proportion of the HZ cases developing PHN was obtained from the study by Pieters et al. [19] based on epidemiological data from the UK General Practice Research Database (GPRD) reported by Gauthier et al. [20].

### 4.14 Proportion of PHN and HZ based on pain severity

The proportions of PHN and HZ based on pain severity was obtained from Pieters et al. [19]. This study computed these values from the UK GPRD database reported by Gauthier et al. [20]. The pain severity levels were categorized into 4 classes: no pain (represented by score 0 based on a scale of 0-10), mild pain (scores 1-2), moderate pain (scores 3-6) and severe pain (scores 7-10). The authors assumed that patients suffering from PHN only experience moderate to severe pain. For more details, see Pieters et al. [19] and Gauthier et al. [20].

### 4.15 Durations of HZ and PHN

The average number of days of the HZ and PHN were obtained from Pieters et al. [19]. These durations were obtained from the NCSF survey. For more details, see Bilcke et al. [16] and Pieters et al. [19].

### 4.16 Quality adjusted life years (QALYs) weights for HZ and PHN

The QALY weights for HZ and PHN where obtained from the study by Pieters et al. [19] who utilized results of van Hoek et al. [21] to calculate the utility weights for the different levels of

pain severity (i.e, mild, moderate and severe pain). See Pieters et al. [19] and Van Hoek et al. [21] for more details on the computation of the utility weights.

### 4.17 Costs of CP treatments

The total costs of hospitalized and ambulatory treated CP patients was obtained from Bilcke et al. [16] and were computed from the National Christian Sickness Fund (NCSF) survey. The survey comprised of the NCSF members and sought information on costs related to CP and HZ, including the direct medical costs due to consultations with GPs and specialists, ambulatory medical expenses, medications, non-medical indirect costs and personal costs. The survey also asked about the duration of the CP and HZ illnesses. The costs were adjusted to the 2023 price level using the ratio of the Consumer Price index (CPI health) of the years 2023 and 2009 in Belgium [31]. The average total cost of an hospitalized CP patient was €3996.05. The average cost of an ambulatory CP patient was €49.97. The average cost for a CP patient not seeking medical care was €0. For more information on the survey and the determination of the costs, see Bilcke et al. [16].

### 4.18 Costs of HZ and PHN treatments

The total direct costs of HZ infection/episode including PHN were obtained from Pieters et al. [19] who utilized data collected from the survey conducted through the NCSF and included a total of 153 hospitalized patients due to HZ and 130 ambulatory HZ patients [16]. The costs were adjusted to the 2023 price level using the ratio of the Consumer Price index (CPI health) of the years 2023 and 2018 in Belgium [31]. For more information see Bilcke et al. [16] and Pieters et al. [19].

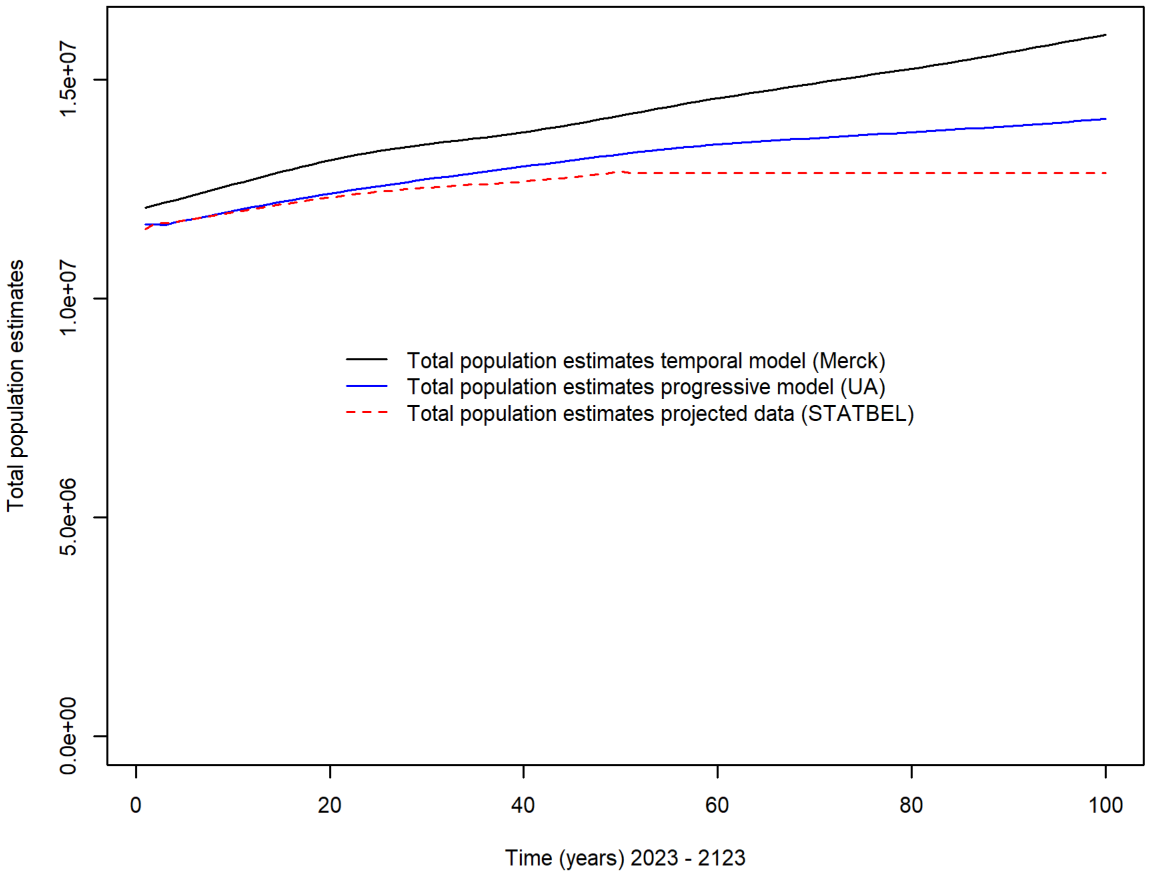


Supplementary Fig 7: Comparison of the total population estimates for the *Temp* and *Prog* models with the predicted estimates from STATBEL.

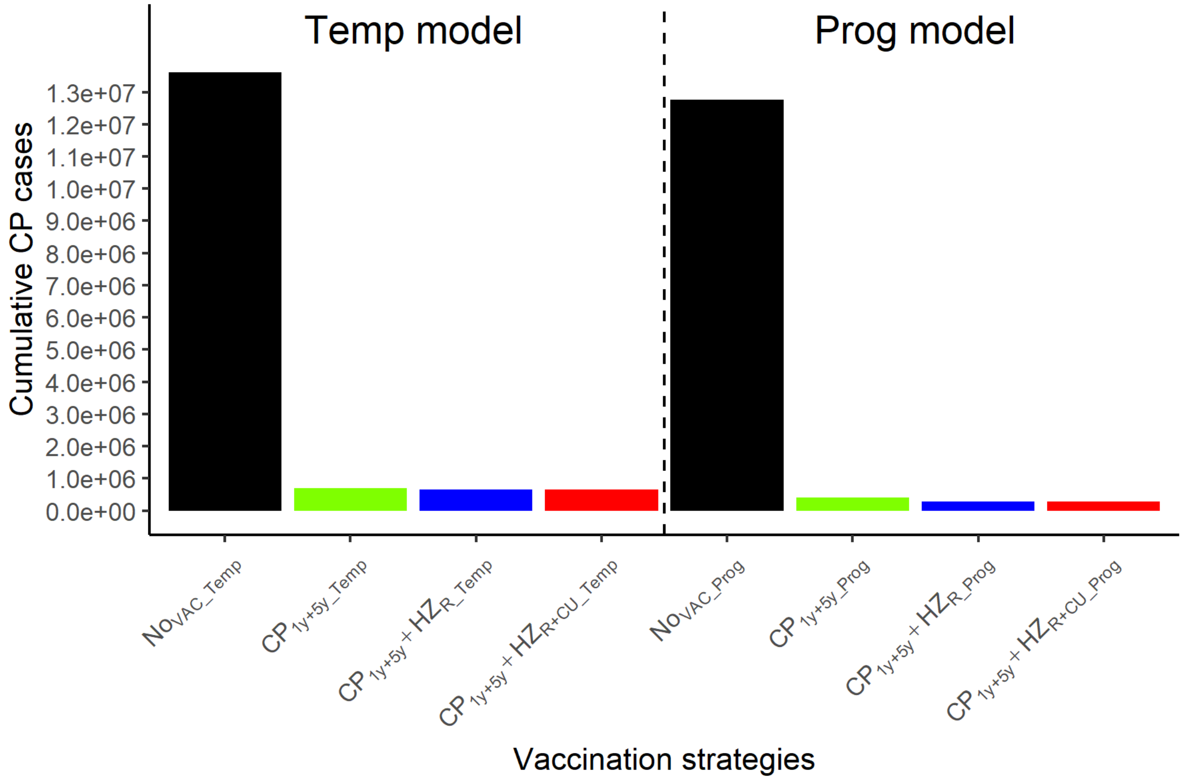


Supplementary Fig 8: Cumulative number of CP cases in 100 years for all the vaccination strategies in the *Temp* and *Prog* models. The vaccination strategies annotated with *Temp* represent the outcomes from the MSD model which employed the temporal immunity boosting assumption while the ones annotated with *Prog* represent the outcomes from the UA model which employed the progressive immunity boosting assumption.

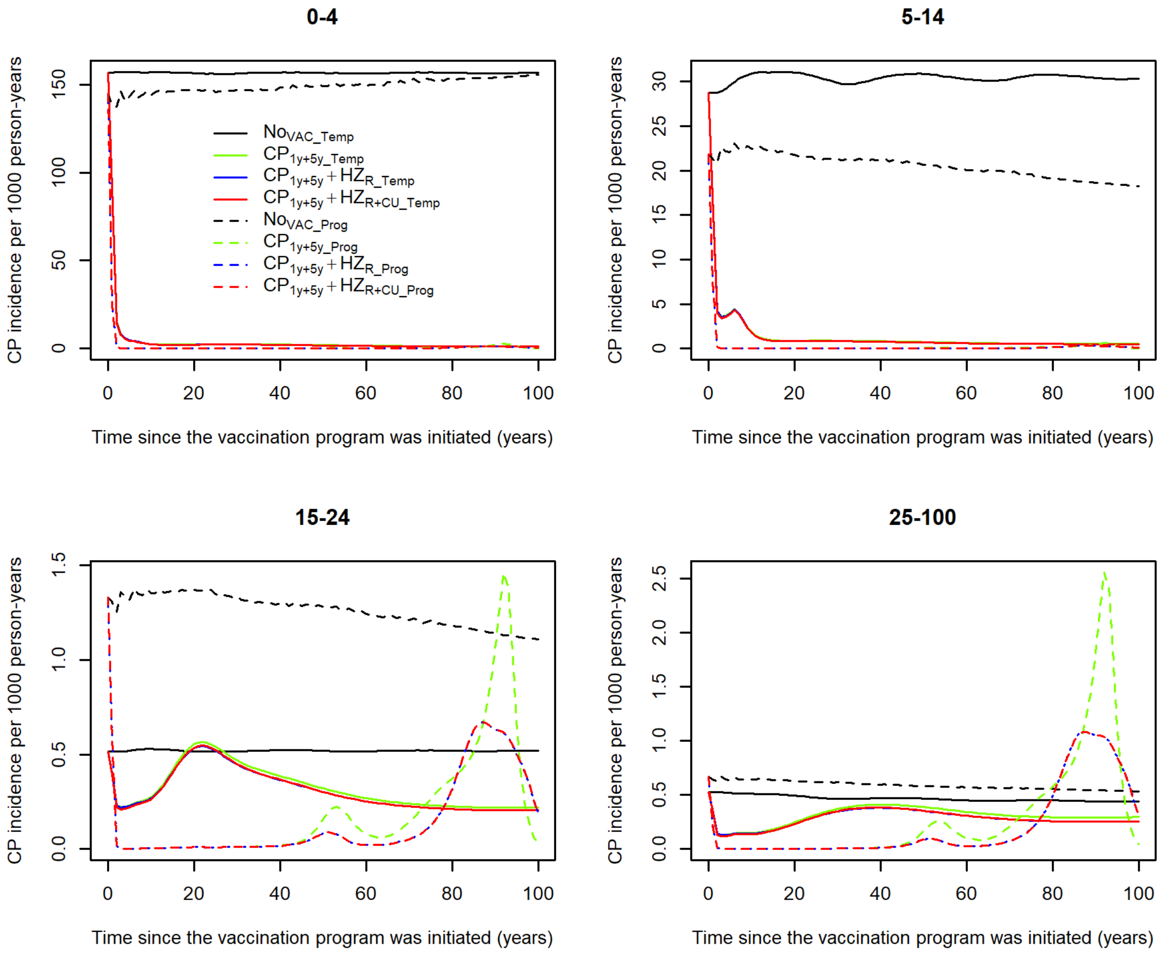


Supplementary Fig 9: Projected age-specific CP incidence in the $Temp$ and $Prog$ models for 100 years. The vaccination strategies annotated with $Temp$ represent the outcomes from the MSD model which employed the temporal immunity boosting assumption while the ones annotated with $Prog$ represent the outcomes from the UA model which employed the progressive immunity boosting assumption.

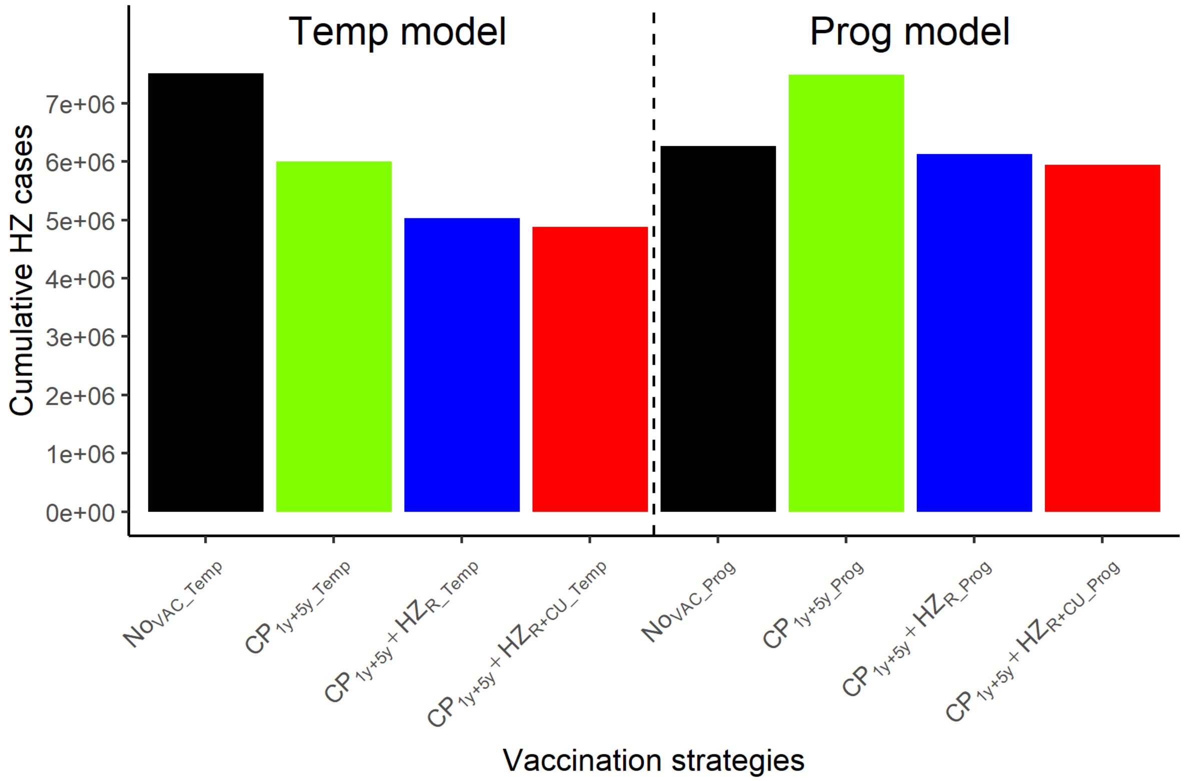


Supplementary Fig 10: Cumulative number of HZ cases in 100 years for all the vaccination strategies in the *Temp* and *Prog* models. The vaccination strategies annotated with *Temp* represent the outcomes from the MSD model which employed the temporal immunity boosting assumption while the ones annotated with *Prog* represent the outcomes from the UA model which employed the progressive immunity boosting assumption.

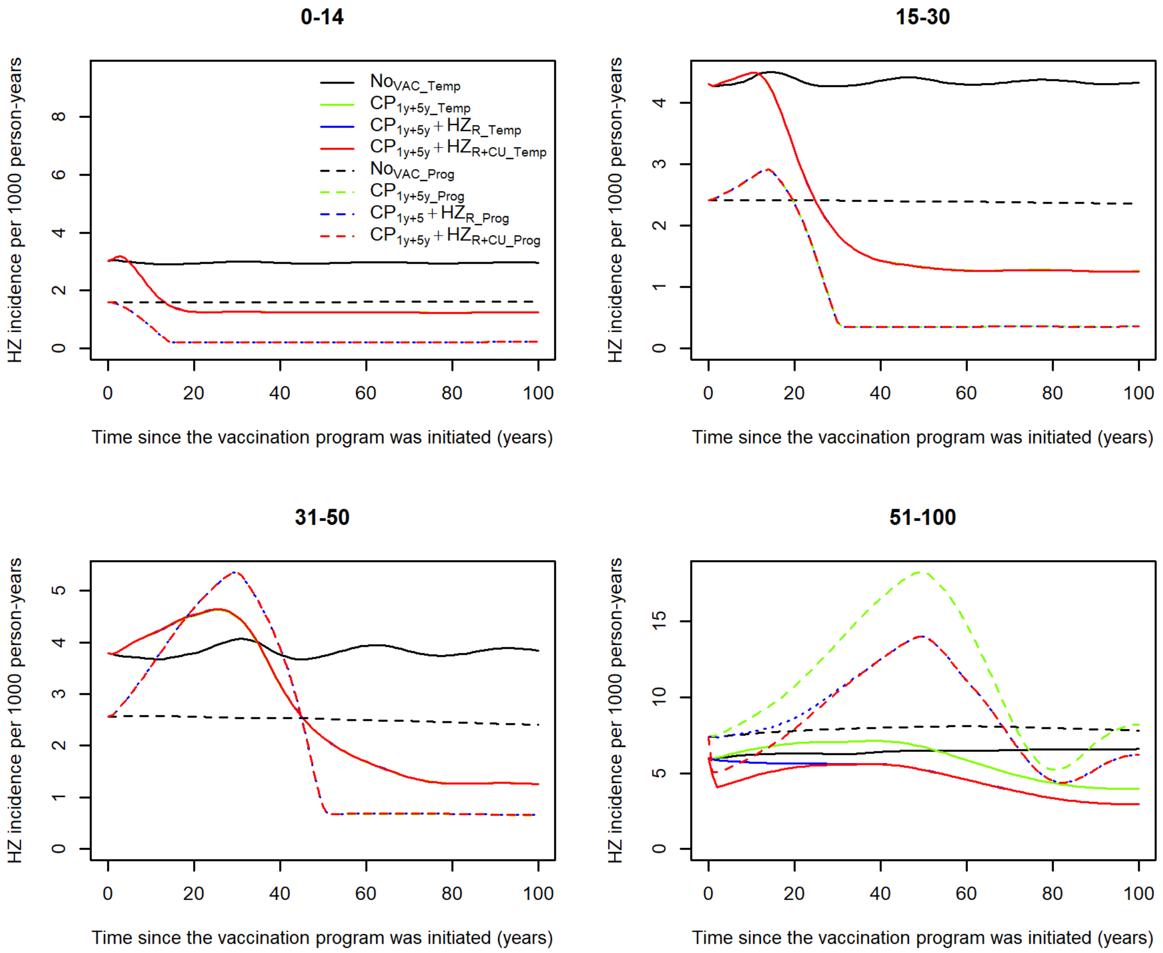


Supplementary Fig 11: Projected age-specific HZ incidence in the *Temp* and *Prog* models for 100 years. The vaccination strategies annotated with *Temp* represent the outcomes from the MSD model which employed the temporal immunity boosting assumption while the ones annotated with *Prog* represent the outcomes from the UA model which employed the progressive immunity boosting assumption. The green solid lines representing the *Temp* model for the age groups 0–14, 15–30, and 31–50 are overlaid by the red and blue lines. Similarly, the dashed green lines representing the *Prog* model are overlaid by the blue and red lines for the same age groups.

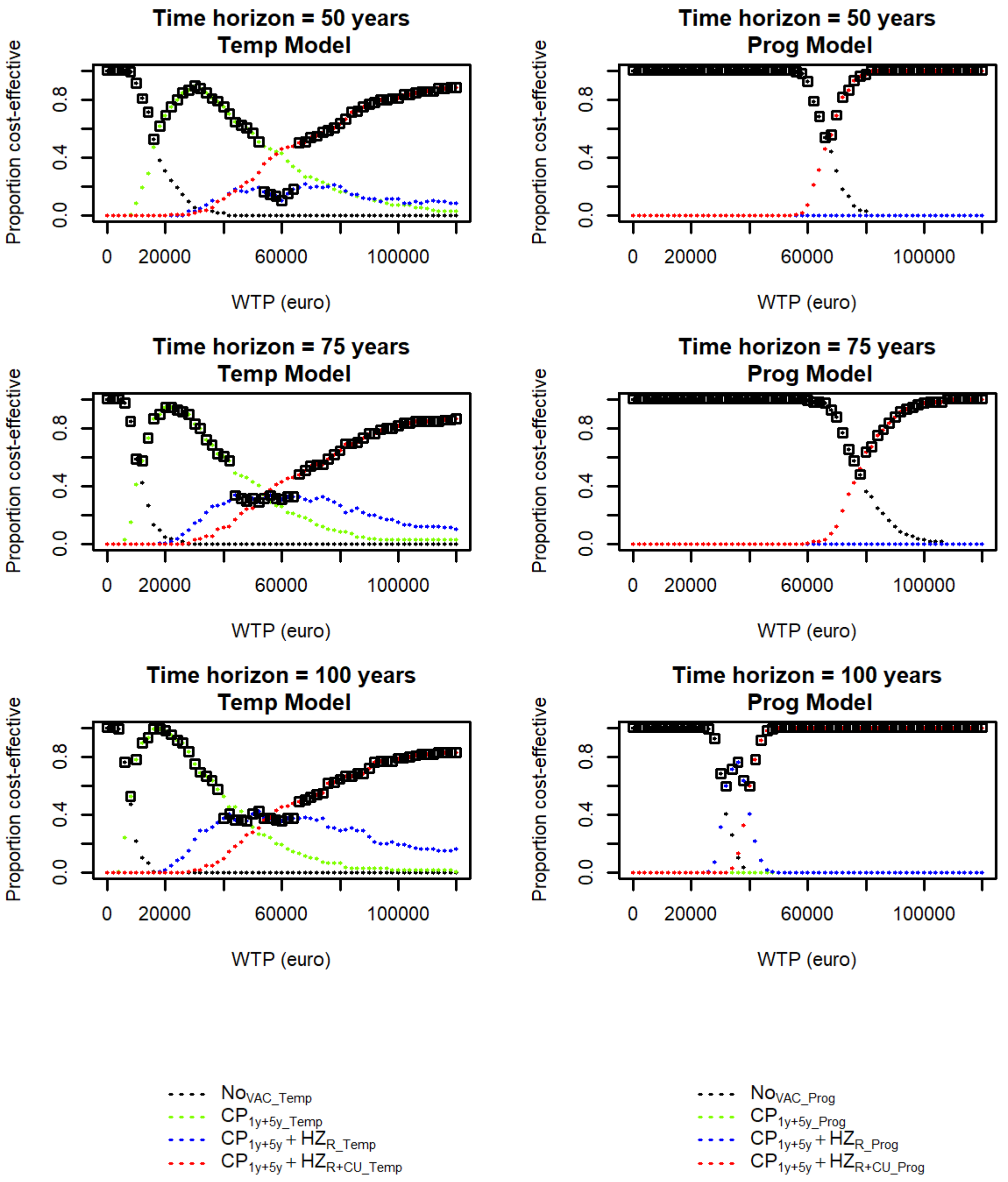


Supplementary Fig 12: Cost-effectiveness acceptability curves for the *Temp* model (left) and *Prog* model (right) showing the probability of each vaccination strategy to be the most cost-effective one, and cost-effectiveness acceptability frontiers showing for the cost-effective strategy only the degree of evidence in favour of that strategy for different values of WTP thresholds values. Discount rates for costs and effects are 3.0% and 1.5%, respectively, with three time horizons considered (TH) (50, 75 and 100 years). Results are based on n = 104 samples drawn with probabilistic sensitivity analysis.

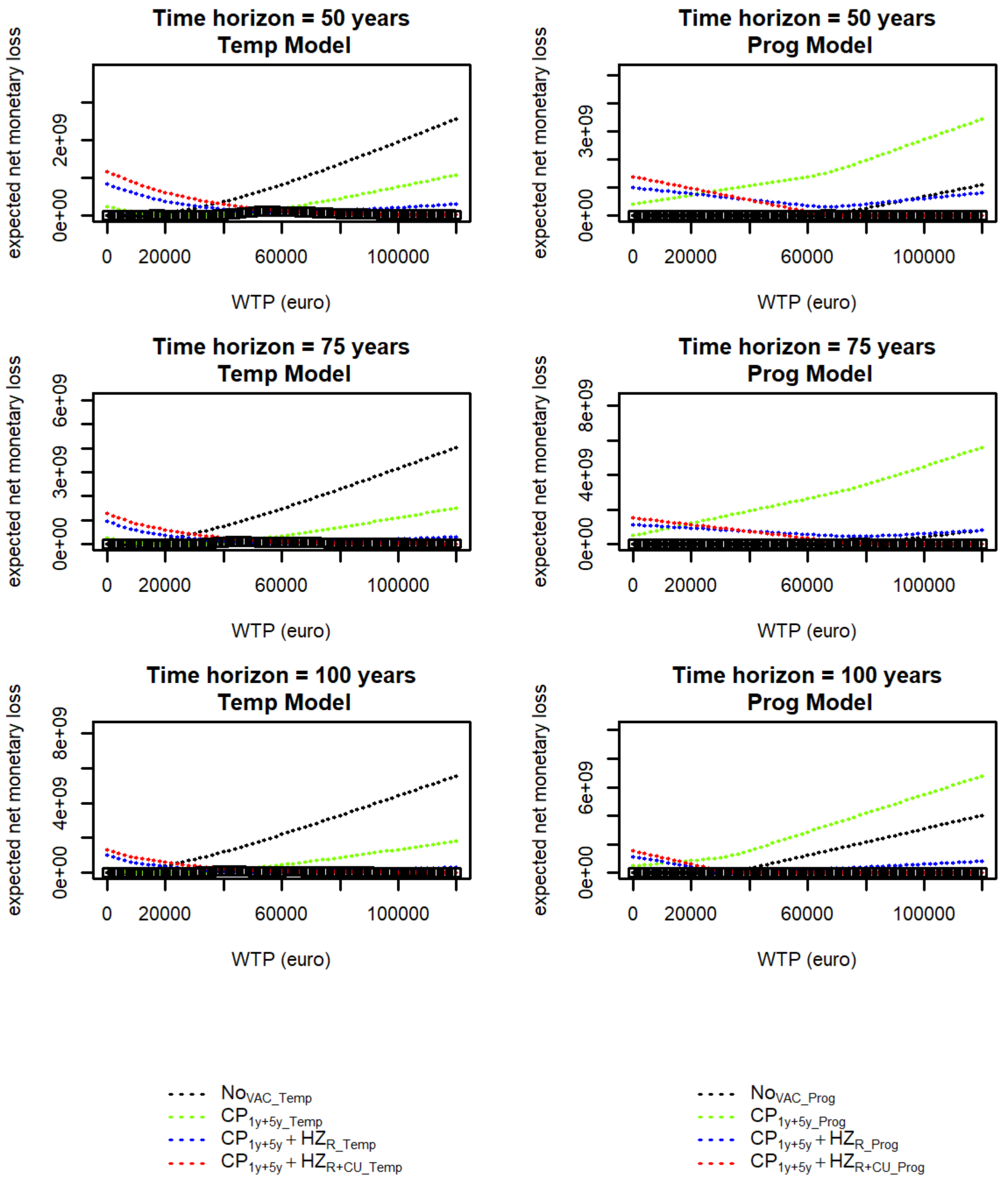


Supplementary Fig 13: The expected net loss curves and the expected net loss acceptability frontier for the *Temp* model (left) and *Prog* model (right) showing for each vaccination strategy, the expected net loss (net loss compared to the cost-effective strategy, averaged over the 104 sampled values during the probabilistic sensitivity analysis) for a range of WTP thresholds. Discount rates for costs and effects are 3.0% and 1.5%, respectively, with three time horizons considered (TH) (50, 75 and 100 years). Results are based on n = 104 samples drawn with probabilistic sensitivity analysis.

| Time horizon = 50 years | | | | | |
|---|---|---|---|---|---|
| Vaccination Strategy | **CP cases** | **HZ cases** | **QALY loss** | **Total costs** | **INMBs** |
| $No_{VAC\_Temp}$ | 6,527,751 | 3,437,667 | 89,774 | 618,209,452 | 0 |
| $No_{VAC\_Prog}$ | 6,187,220 | 2,933,853 | 132,871 | 681,000,075 | 0 |
| $CP_{1y+5y\_Temp}$ | 411,883 | 3,405,416 | 75,494 | 852,978,961 | 336,450,525 |
| $CP_{1y+5y\_Prog}$ | 37,943 | 4,298,214 | 148,849 | 1,101,039,231 | -1,059,194,501 |
| $CP_{1y+5y} + HZ_{R\_Temp}$ | 393,260 | 2,936,449 | 64,091 | 1,448,649,362 | 196,878,039 |
| $CP_{1y+5y} + HZ_{R\_Prog}$ | 35006 | 3,584,598 | 122,148 | 1,679,642,523 | -569,728,155 |
| $CP_{1y+5y} + HZ_{R+CU\_Temp}$ | 390,826 | 2,796,564 | 58,927 | 1,781,072,712 | 70,991,843 |
| $CP_{1y+5y} + HZ_{R+CU\_Prog}$ | 34,959 | 3,409,126 | 112,087 | 2,072,317,639 | -559,921,534 |
| **Time horizon = 75 years** | | | | | |
| $No_{VAC\_Temp}$ | 9,983,789 | 5,406,115 | 120,190 | 735,294,729 | 0 |
| $No_{VAC\_Prog}$ | 9,422,322 | 4,596,086 | 204,384 | 848,639,444 | 0 |
| $CP_{1y+5y\_Temp}$ | 560,276 | 4,834,263 | 97,049 | 993,540,844 | 667,394,378 |
| $CP_{1y+5y\_Prog}$ | 97,527 | 6,353,415 | 240,004 | 1,347,735,726 | -1,923,908,421 |
| $CP_{1y+5y} + HZ_{R\_Temp}$ | 527,387 | 4,097,621 | 81,247 | 1,683,214,556 | 609,778,641 |
| $CP_{1y+5y} + HZ_{R\_Prog}$ | 55,973 | 5,211,676 | 194,952 | 1,979,462,381 | -753,537,253 |
| $CP_{1y+5y} + HZ_{R+CU\_Temp}$ | 525,176 | 3,957,438 | 76,109 | 2,015,610,146 | 482,905,810 |
| $CP_{1y+5y} + HZ_{R+CU\_Prog}$ | 55,929 | 5,036,100 | 184,884 | 2,372,129,154 | -743,513,666 |
| **Time horizon = 100 years** | | | | | |
| $No_{VAC\_Temp}$ | 13,611,750 | 7,511,947 | 142,909 | 795,630,845 | 0 |
| $No_{VAC\_Prog}$ | 12,763,980 | 6,267,590 | 269,733 | 982,846,463 | 0 |
| $CP_{1y+5y\_Temp}$ | 695,472 | 5,996,726 | 109,588 | 1,055,619,645 | 1,072,866,295 |
| $CP_{1y+5y\_Prog}$ | 399,546 | 7,491,975 | 288,834 | 1,464,643,258 | -1,245,866,812 |
| $CP_{1y+5y} + HZ_{R\_Temp}$ | 647,372 | 5,026,592 | 90,971 | 1,798,654,031 | 1,074,506,094 |
| $CP_{1y+5y} + HZ_{R\_Prog}$ | 290,259 | 6,126,466 | 233,543 | 2,129,251,331 | 301,172,989 |
| $CP_{1y+5y} + HZ_{R+CU\_Temp}$ | 645,202 | 4,886,215 | 85,851 | 2,131,024,988 | 946,924,477 |
| $CP_{1y+5y} + HZ_{R+CU\_Prog}$ | 290,259 | 5,950,738 | 223,469 | 2,521,907,487 | 311,493,304 |

Supplementary Table 8: Cumulative number of CP cases, HZ cases, QALY loss, treatment and intervention costs and incremental net monetary benefits by each vaccination strategy and time horizon (TH = 50, 75 & 100 years). All outcomes are reported with 3.0% discount rate for costs and 1.5% discount rate for effects.

# References


[1] van Lier A, Lugnér A, Opstelten W, Jochemsen P, Wallinga J, Schellevis F, et al. Distribution of health effects and cost-effectiveness of varicella vaccination are shaped by the impact on herpes zoster. EBioMedicine. 2015;2(10):1494-9.

[2] Age specific prospective mortality rate data in Belgium from STATBEL (statistics Belgium);. https://statbel.fgov.be/nl.

[3] United Nations, Department of Economic and Social Affairs, Population Division (2022). World Population Prospects 2022;. https://population.un.org/wpp/.

[4] Riffe T, Aburto J, Alexander M, Fennell S, Kashnitsky I, Pascariu M, et al.. DemoTools: An R package of tools for aggregate demographic analysis; 2019. URL: https://github.com/timriffe/DemoTools/.

[5] Rogers A, Castro LJ. Model migration schedules. 1981.

[6] Seward JF, Zhang JX, Maupin TJ, Mascola L, Jumaan AO. Contagiousness of varicella in vaccinated cases: a household contact study. Jama. 2004;292(6):704-8.

[7] Civen R, Chaves SS, Jumaan A, Wu H, Mascola L, Gargiullo P, et al. The incidence and clinical characteristics of herpes zoster among children and adolescents after implementation of varicella vaccination. The Pediatric Infectious Disease Journal. 2009;28(11):954-9.

[8] Byrd RH, Lu P, Nocedal J, Zhu C. A limited memory algorithm for bound constrained optimization. SIAM Journal on Scientific Computing. 1995;16(5):1190-208.

[9] Pawaskar M, Siddiqui MK, Takyar J, Sharma A, Fergie J. Relative efficacy of varicella vaccines: network meta-analysis of randomized controlled trials. Current Medical Research and Opinion. 2022:1-11.

[10] Gershon AA, Raker R, Steinberg S, Topf-Olstein B, Drusin LM. Antibody to varicella-zoster virus in parturient women and their offspring during the first year of life. Pediatrics. 1976;58(5):692-6.

[11] Cunningham AL, Lal H, Kovac M, Chlibek R, Hwang SJ, Díez-Domingo J, et al. Efficacy of the herpes zoster subunit vaccine in adults 70 years of age or older. New England Journal of Medicine. 2016;375(11):1019-32.

[12] Boutry C, Hastie A, Diez-Domingo J, Tinoco JC, Yu CJ, Andrews C, et al. The adjuvanted recombinant zoster vaccine confers long-term protection against herpes zoster: interim results of an extension study of the pivotal phase 3 clinical trials ZOE-50 and ZOE-70. Clinical Infectious Diseases. 2022;74(8):1459-67.

[13] Izurieta HS, Strebel PM, Blake PA. Postlicensure effectiveness of varicella vaccine during an outbreak in a child care center. Jama. 1997;278(18):1495-9.

[14] Gordon J, Meader F, et al. The Period of Infectivity and Serum Prevention of Chicken-pox. Journal of the American Medical Association. 1929;93:2013-5.

[15] Schuette MC, Hethcote HW. Modeling the effects of varicella vaccination programs on the incidence of chickenpox and shingles. Bulletin of Mathematical Biology. 1999;61:1031-64.

[16] Bilcke J, Ogunjimi B, Marais C, De Smet F, Callens M, Callaert K, et al. The health and economic burden of chickenpox and herpes zoster in Belgium. Epidemiology & Infection. 2012;140(11):2096-109.

[17] Bilcke J, Jan van Hoek A, Beutels P. Childhood varicella-zoster virus vaccination in Belgium: cost-effective only in the long run or without exogenous boosting? Human Vaccines & Immunotherapeutics. 2013;9(4):812-22.

[18] Rodrigues F, Marlow R, Gouveia C, Correia P, Brett A, Silva C, et al. Prospective study of loss of health-related quality adjusted life years in children and their families due to uncomplicated and hospitalised varicella. Vaccine. 2023;41(6):1182-9.

[19] Pieters Z, Ogunjimi B, Beutels P, Bilcke J. Cost-effectiveness analysis of herpes zoster vaccination in 50-to 85-year-old immunocompetent Belgian cohorts: a comparison between no vaccination, the adjuvanted subunit vaccine, and live-attenuated vaccine. Pharmacoeconomics. 2022;40(4):461-76.

[20] Gauthier A, Breuer J, Carrington D, Martin M, Rémy V. Epidemiology and cost of herpes zoster and post-herpetic neuralgia in the United Kingdom. Epidemiology & Infection. 2009;137(1):38-47.

[21] Van Hoek AJ, Gay N, Melegaro A, Opstelten W, Edmunds W. Estimating the cost-effectiveness of vaccination against herpes zoster in England and Wales. Vaccine. 2009;27(9):1454-67.

[22] Sharomi O, Xausa I, Nachbar R, Pillsbury M, Matthews I, Petigara T, et al. Modeling the Impact of Exogenous Boosting and Universal Varicella Vaccination on the Clinical and Economic Burden of Varicella and Herpes Zoster in a Dynamic Population for England and Wales. Vaccines. 2022;10(9):1416.

[23] Gao F, Han L. Implementing the Nelder-Mead simplex algorithm with adaptive parameters. Computational Optimization and Applications. 2012;51(1):259-77.

[24] Poletti P, Melegaro A, Ajelli M, Del Fava E, Guzzetta G, Faustini L, et al. Perspectives on the impact of varicella immunization on herpes zoster. A model-based evaluation from three European countries. PloS One. 2013;8(4):e60732.

[25] Trollor J. Herpes zoster in general practice. Australian Family Physician. 1987;16(8):1133-7.

[26] Forbes H, Douglas I, Finn A, Breuer J, Bhaskaran K, Smeeth L, et al. Risk of herpes zoster after exposure to varicella to explore the exogenous boosting hypothesis: self controlled case series study using UK electronic healthcare data. BMJ. 2020;368.

[27] Riera-Montes M, Bollaerts K, Heininger U, Hens N, Gabutti G, Gil A, et al. Estimation of the burden of varicella in Europe before the introduction of universal childhood immunization. BMC Infectious Diseases. 2017;17:1-16.

[28] Mossong J, Hens N, Friederichs V, Davidkin I, Broman M, Litwinska B, et al. Parvovirus B19 infection in five European countries: seroepidemiology, force of infection and maternal risk of infection. Epidemiology & Infection. 2008;136(8):1059-68.

[29] Hoang TV, Coletti P, Kifle YW, Kerckhove KV, Vercruysse S, Willem L, et al. Close contact infection dynamics over time: insights from a second large-scale social contact survey in Flanders, Belgium, in 2010-2011. BMC Infectious Diseases. 2021;21(1):1-15.

[30] Coplan PM, Schmader K, Nikas A, Chan IS, Choo P, Levin MJ, et al. Development of a measure of the burden of pain due to herpes zoster and postherpetic neuralgia for prevention trials: adaptation of the brief pain inventory. The Journal of Pain. 2004;5(6):344-56.

[31] Belgian Consumer Price Index, Author = Statbel, Year = 2023, note = URL: https://statbel.fgov.be/en/themes/consumer-prices/consumer-price-index#figures;.
