# Supplementary material for: Comparative analysis of the impact of chickenpox and herpes zoster vaccination in Belgium under two different exogenous boosting mechanisms
Source: PLoS One. 2026 Mar 19;21(3):e0342896. doi: 10.1371/journal.pone.0342896 (PMC13001986; doi:10.1371/journal.pone.0342896)
Supplement: Attachment — Submitted filename: Response to Reviewers.pdf [file pone.0342896.s002.pdf]

Point-by-point reply for the manuscript with submission ID [PONE-D-25-45382] - [EMID:ce9f0695f8d35f8d]

Thank you for the opportunity to revise our manuscript entitled “Comparative analysis of the impact of chickenpox and herpes zoster vaccination in Belgium under two different exogenous boosting mechanisms”. We feel that we have adequately addressed all issues raised by the reviewers and we hope you are willing to consider our revised manuscript for publication in Plos One.

Hereunder, we have provided a detailed point-by-point reply to all comments raised by the reviewers together with a description of the changes we made in the revised manuscript (using red and blue for added and deleted parts, respectively, in the annotated version of the manuscript). The Figure numbers and citations in this point by point response follow an independent enumeration with respect to the main manuscript.

Reviewer 1 Comments:
This manuscript includes analyses of varicella and herpes zoster vaccination strategies in the country of Belgium, in order to determine the various effects on herpes zoster burden. The authors observed that the impact varied significantly depending on the assumed boosting mechanism of immunity to VZV over the lifespan of an individual. The manuscript is filled with data, including considerable Supplemental Data. However, the interpretations of the data are not always clearly delineated within the manuscript. Further the costs of herpes zoster vaccination are buried within the Supplemental Data. The costs of vaccination should be clearly stated in the main text of the manuscript. See comments below.

We thank the reviewer for taking their time to review our work and for thoughtful comments and suggestions as well as appreciating our work.

1.Title. To this Reviewer, it seems peculiar to call the varicella vaccine by the name of chickenpox vaccine? The Merck Company even named their vaccine as Varivax (not chickenvax).

We thank the reviewer for pointing this out. We have now specified the vaccines used against chickenpox (i.e., Varivax and ProQuad).

2.Herpes zoster vaccine in title and everywhere in the manuscript. The are 2 herpes zoster vaccines: the live vaccine (Zostavax) and the recombinant vaccine Shingrix (often called RZV). From the title, it is unclear which vaccine is being examined. Shingrix only cited later in line 176 of Methods. Suggest adding the word recombinant zoster vaccine into the title to avoid confusion.

We thank the reviewer for this helpful comment and suggestion. We agree that there are two herpes zoster vaccines: the live attenuated vaccine (Zostavax) by Merck and the recombinant zoster vaccine (RZV), Shingrix, by GSK. However, Zostavax has been discontinued globally, and Merck has permanently stopped its marketing and production. In the European Union, its marketing authorization was officially withdrawn on June 1, 2025. As

a result, the Recombinant zoster vaccine, Shingrix, is currently the only HZ vaccine authorized and available on the market.

Regarding the suggestion to include the recombinant zoster vaccine in the title, we believe doing so would make the title unnecessarily very long. Nonetheless, to ensure clarity about the specific vaccine considered, we have added the following information in the abstract, Methods subsection: *"The HZ vaccine considered was the recombinant zoster vaccine (RZV), Shingrix."* Additionally, in the final paragraph of the Background section, we have included the sentence: *"Throughout this work, we consider the CP vaccines (Varivax and ProQuad) by Merck & Co., Inc., Rahway, NJ, USA (MSD), and the recombinant zoster vaccine, Shingrix, by GlaxoSmithKline SA, Belgium."*

3.Abstract. Likewise, in Abstract, add the word recombinant to zoster vaccine.

We thank the reviewer for this suggestion. We have added the word recombinant to zoster vaccine in the abstract methods subsection.

4.Method. The title states that the study uses data from Belgium. Restate that point at the front of Methods. Also give us basic data about Belgium: the average life span, total population, percent of population under 18 years, and percent of population over 65 years.

We thank the reviewer for this suggestion. We have added the following information in the Methods section 'Both teams adapted their models to the Belgian context with common input data. Belgium, located in Northwestern Europe, has a population of approximately 11.8 million and an average life expectancy at birth of 82.4 years (2024). The population age structure consists of 16.7% under 15 years, 63.9% between 15 and 64 years, and 19.4% aged 65 years and above [1].'

5.Methods, line 171. Add the word recombinant zoster vaccine for clarity.

We thank the reviewer for this suggestion. We have added the word 'recombinant zoster vaccine' as suggested by the reviewer.

6.Methods. New section needed. Section 4.19 in Supplemental data called Vaccine Pricing. THESE DATA ARE CRITICAL. Move this entire paragraph on vaccine costs into the main text of Methods as a new section.

We thank the reviewer for this comment and suggestion. We have added a new subsubsection 2.2.1 in the Methods section, data description subsection titled 'Vaccine pricing and cost of administration', with the following information, 'The costs of vaccines were obtained from the Belgian Center for Pharmacotherapeutic report [2] indicating a private sector price per dose and is as follows: recombinant zoster vaccine (GSK) - € 170.26; Varivax (MSD) - € 52.52; ProQuad (MSD) - € 73.69. The costs for the CP vaccine administration per dose was taken to be € 18.16, whilst that for the recombinant zoster

vaccine was taken to be € 30.0, based on the tariff for an accredited GP consultation in Belgium [3].'

7.Discussion. Line 323. Clearly re-state in the first few sentences of the Discussion that this analysis pertains only to the country of Belgium. Or if that statement is not true, clarify whether this analysis can be applied to other countries, and which other countries. The life span in Belgium is around 82 years. By comparison, the life span in at least 10 countries in Africa is under 60 years.

We thank the reviewer for this comment and suggestion. We have added the words 'in Belgium' in the first sentence of the discussion section (line 290) to reiterate that the analysis considered the Belgian setting/context. In terms of whether this analysis can be applied to other countries or not, we need to stress that this comparative modeling primarily aimed to provide more insights on the uncertainty surrounding policy making regarding the implementation of universal CP and HZ vaccination programs based on cost-effectiveness outcomes. This uncertainty is majorly due to the lack of understanding of the exact biological mechanisms underlying the pathogenesis of the HZ. To date, the exogenous boosting hypothesis postulated back in 1965 remains the only mechanism to model the reactivation of the latent varicella zoster virus (VZV) to HZ. However, it is important to note that the mechanism is still widely debated due to the scientific uncertainty underlying it. Thus, in a broader and general perspective, this study which qualitatively and quantitatively compared results from the two main exogenous boosting mechanisms widely adopted up to date, aimed to provide more insights to policy makers in countries considering to implement these universal vaccination programs that effectiveness outcomes are mainly dependent on the adapted exogenous boosting mechanism and that this can lead to significant differences in the cost-effectiveness outcomes.

8.Major discrepancy with a published article. In the Supplemental data, the authors state that the cost of Shingrix is 170 (vaccine) + 30 (administration) = 200 Euros. In a paper in the journal VACCINE (Vol 30:675, 2012; PMID: 22120193), Bilcke et al state the cost of a zoster vaccine must be less than 45 Euros to be cost-effective in Belgium. Please cite this paper in the Discussion and explain the different costs and values for QALY in the 2 reports.

We thank the reviewer for this helpful comment and for suggesting a comparison with the work by Bilcke et al. (2012) [4]. Their study assessed the cost-effectiveness of HZ vaccination among adults aged 60 years and above in Belgium using a static cohort model and the Zostavax vaccine. Their primary objective was to identify the most cost-effective age for HZ vaccination, concluding that vaccination of adults aged 60–64 years could be cost-effective if the Zostavax price was reduced to €45 per dose.

In contrast, our study employed two deterministic dynamic compartmental models incorporating different exogenous boosting mechanisms to capture the relationship between CP and HZ across ages 0–99 years. Our primary aim was to qualitatively and quantitatively compare the effectiveness and cost-effectiveness outcomes of these two modelling frameworks under both CP and HZ vaccination strategies.

We note several fundamental methodological differences that make direct comparison of estimates such as costs and QALYs between the two studies inappropriate. First, we employed a dynamic modeling framework, whereas they used a static cohort model. Second, we modeled both CP and HZ, while their study focused solely on HZ. Third, we considered the full age range (0–99 years), whereas they focused on adults aged 60 years and above. Finally, our study utilized the recombinant zoster vaccine, with an efficacy of 91.3% and an average duration of protection of 49 years, whereas their study used the Zostavax vaccine, which had a shorter duration of protection (less than 10 years) and relatively lower age-specific vaccine efficacies depending on the functional forms of the vaccine efficacy models applied in their modeling framework. For these reasons, we believe that adding a detailed comparison with Bilcke et al. (2012) [4] would not substantially enhance the interpretation of our findings regarding the differences between the two dynamic exogenous boosting models.

However, we note that the later work by Bilcke et al. (2013) [5], which evaluated universal childhood CP vaccination in Belgium and explicitly examined the role of the exogenous boosting mechanism while also exploring adult HZ vaccination programs, is directly relevant to our study. We have therefore incorporated this study in our discussion accordingly.

9.Abstract. The cost of vaccination is a critical component of this analysis. Please add the cost of vaccination into the Abstract of this manuscript.

We thank the reviewer for this suggestion to add the cost of vaccination in the abstract of this manuscript. As per the reviewers suggestion, we have added the following sentence in the methods part of the abstract. 'The vaccine prices per dose were as follows: Varivax - € 52.52, ProQuad - € 73.69, recombinant zoster vaccine - € 170.26.'

10.Conclusion. Again, please clearly state in the Conclusion whether the analysis in this report refers only to Belgium (see comment 7). Avoid use of words such as structural assumptions; say what you mean precisely. Overall, the Conclusion is a weak statement.

We thank the reviewer for this comment. Regarding the suggestion to clearly state in the conclusion whether the analysis in this report refers only to Belgium, we note that although the two modelling frameworks applied data from Belgium, the overarching aim—to generate insights on the effectiveness and cost-effectiveness of CP and HZ vaccination programs under two widely adopted exogenous boosting mechanisms—is relevant beyond the Belgian context. These findings are also applicable to other countries considering the introduction of universal CP and HZ vaccination programs, particularly given the existing uncertainties surrounding the exact biological and immunological processes underlying VZV reactivation to HZ. It is for this reason that the conclusion is formulated from a broader, more general perspective.

Based on the reviewer's suggestion to avoid using the word 'structural', we have revised the sentence in the conclusion. The updated sentence now reads: 'Policy makers should be aware that model predictions for CP and HZ may depend heavily on the exogenous boosting

mechanism adopted, which should be taken into account when evaluating CP and HZ vaccination policies.

Reviewer #2: The article by Wambua and colleagues is a comparative analysis of chickenpox and herpes zoster vaccination in Belgium under two different exogenous boosting mechanisms. This work is important as universal chickenpox and herpes zoster present a major public health problem. The impact of vaccination varies depending on the assumed boosting mechanism. The paper presents a cost-utility analysis on two deterministic compartmental models each employing a different boosting mechanism (temporal or progressive immunity). The models, which have been developed by different teams, investigated the epidemiological impact and cost-effectiveness. The article reported that universal chickenpox vaccination can significantly reduce chickenpox disease burden. However, the impact on herpes zoster disease varies depending on the assumed boosting mechanism. Although the paper includes some technical details in the main text, it is well written. The model is extensive and well described. I have some concerns related to the economic analysis.

We thank the reviewer for accepting and taking their time to review our work, for their valuable comments and suggestions and for appreciation of our work.

Major comments
1. The analysis only use fixed values for costs and QALYs. The paper should investigate the impact of the various costs and QALYs using a sensitivity analysis in which the chosen costs and QALYs are varied with e.g. 50%. In my view this is important as for instance the some of the costs are taken from a paper published in 2012 (reference 16 in the supplement). It is unlikely that the same costs have to be paid in the present day. I therefore believe it is important to include a sensitivity analysis in which these costs are increased (this could for instance be done using tornado plots).

2. Similarly, the QALY scores that are used are from older people whereas the models include the entire life span (these scores are from references 19 and 21 in the supplement). Reference 16 uses different scores. As QALY scores are key for cost-effectiveness, it is important to show the impact of varying these scores on the main outcome.

We thank the reviewer for the comments and helpful suggestions. Regarding the costs and QALYs used in our analysis, we agree with the reviewer that these values were taken from previous studies. For the cost inputs, we adjusted the values to 2023 price levels using the ratio of the consumer price index (CPI health) for 2023 relative to 2009 for costs related to CP treatments [6], and the ratio of 2023 to 2018 for costs related to HZ and postherpetic neuralgia (PHN) treatments [7]. Additional details are provided in Subsections 4.17 and 4.18 of the S1 file.

With respect to the use of fixed values for costs and QALYs in this analysis, we would like to clarify that uncertainty in these inputs was fully accounted for through a probabilistic sensitivity analysis (PSA). The PSA is described in Subsection 2.6 (Health economic

framework), and the probability distributions applied to the cost and QALY inputs are presented in Supplementary Tables 3 and 7 of the S1 file. Thus, our analysis incorporates uncertainty by estimating expected incremental monetary benefits (INMBs) based on averages across multiple sets of input values sampled from these distributions.

3. Please add the time horizon and discounting to the abstract as these are important for the outcomes.

We thank the reviewer for this suggestion. We have added the following information in the methods part of the abstract: ‘Costs and health outcomes were discounted at 3.0% and 1.5% annually, respectively. We used 3 time horizons (i.e., 50, 75 & 100 years) and implemented the healthcare payer perspective throughout the analysis.’

Minor comments

1. Please add a label to the y-axis of Figures 3 and 4. Please also use the same scale for all y-axes in Figures 3 and 4.

We thank the reviewer for the helpful suggestion to add a label to the y-axis of Figures 3 and 4. We have now included the unit “Euros” in the y-axis labels, which now read **INMB (Euros)**.

Regarding the recommendation to use the same scale for y-axes scales for Figures 3 and 4, we explored this option. Although applying a uniform scale is possible, it substantially reduces the visibility of differences in several subplots, particularly those where the differences are small (see Figures 1 and 2 below, corresponding to Figures 3 and 4 of the main text, respectively). Thus, to preserve the visibility of the differences across the subplots, we have opted to retain subplot-specific y-axis scales as it provides clearer visualization.

2. Supplement section 1.1 includes the following sentence: “The risk of HZ reactivation is modeled using the progressive immunity boosting mechanism as described in Subsection ?? in the main text.” Please replace ?? with the appropriate sub-section.

We thank the reviewer for pointing out this. The ?? have been replaced with 2.1 and now the sentence reads ‘The risk of HZ reactivation is modeled using the progressive immunity boosting mechanism as described in Subsection 2.1 in the main text’.

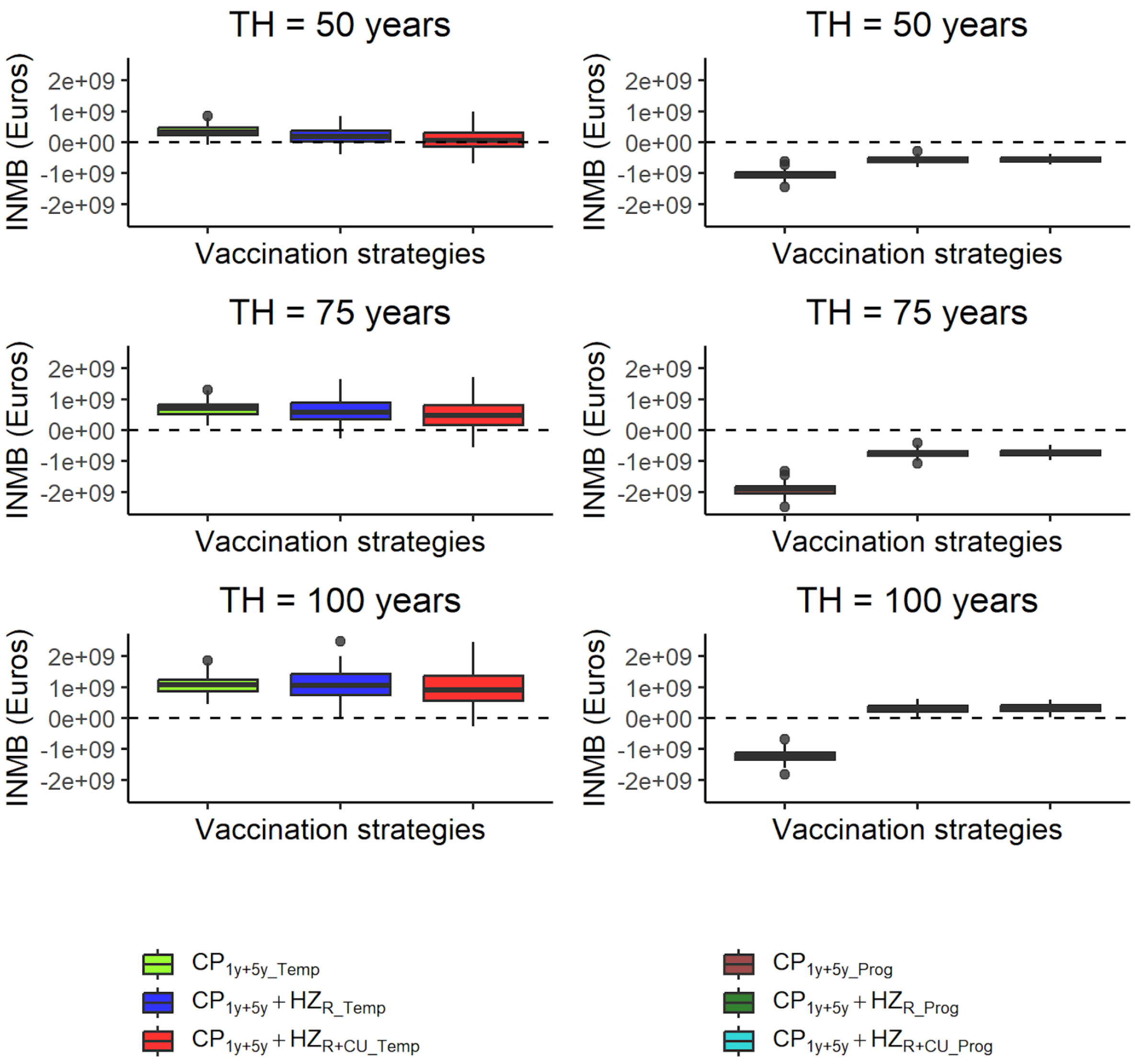

Figure 1: Box plots for incremental net monetary benefits (INMBs) for the Temp model (left) and the Prog model (right) with 2.5%, 25%, 50%, 75% and 97.5% quantiles obtained under probabilistic sensitivity analysis, by inter-strategy comparisons, using a WTP threshold of Euro 40,000 per QALY gained. Three time horizons TH = 50, 75 & 100 years, are considered.

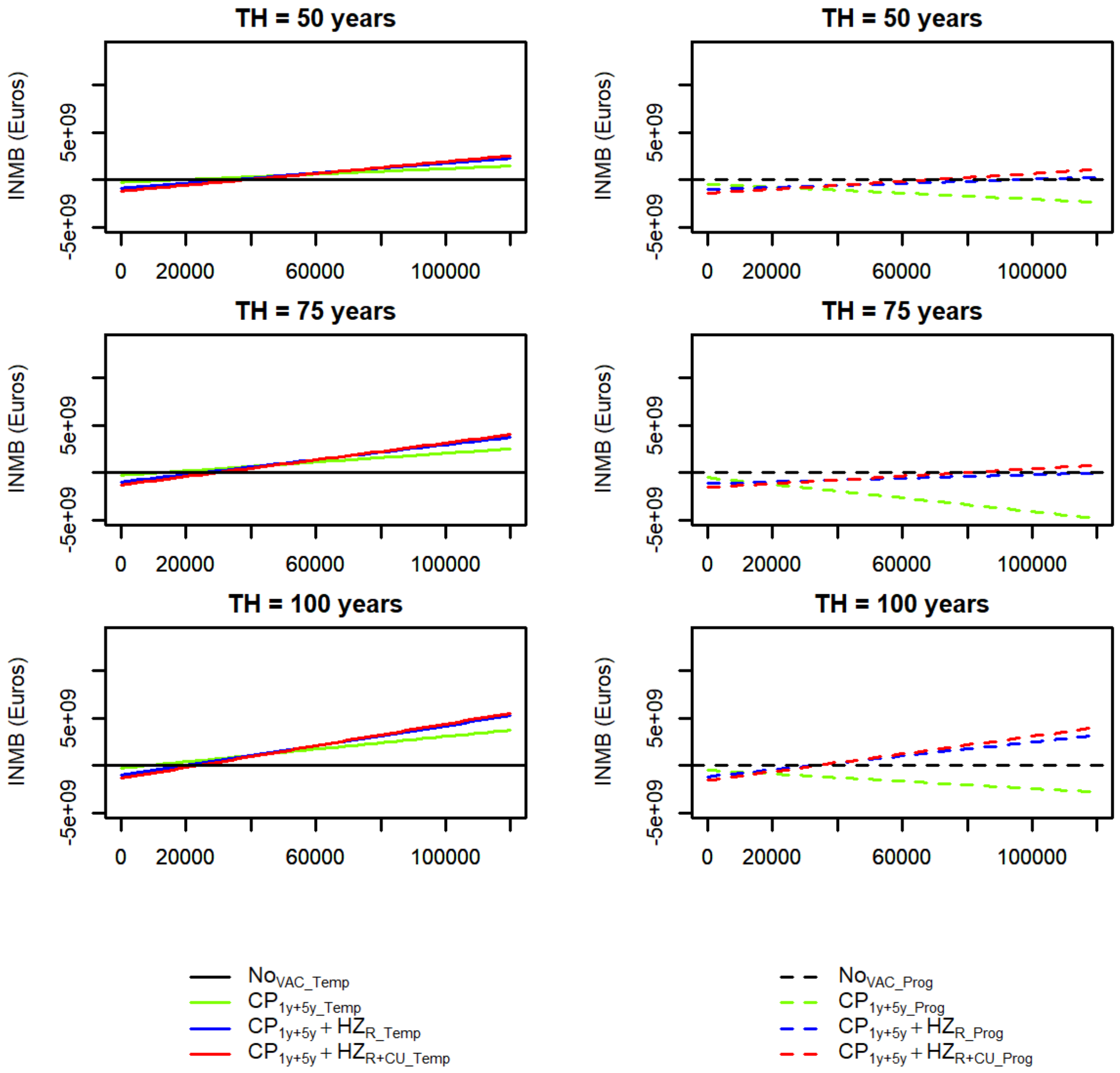

Figure 1: Expected incremental net benefit (INMBs) for the Temp model (left) and the Prog model (right) for the different vaccination strategies obtained under probabilistic sensitivity analysis, by inter-strategy comparisons over a range of WTP per QALY values and three time horizons (TH) (50, 75 and 100 years).

References

1. Age specific population estimates, life expectancy, and age specific prospective mortality rate data in Belgium from STATBEL (statistics Belgium);. https://statbel.fgov.be/nl.
2. Belgian Center for Pharmacotherapeutic Information. Vaccine against zona. 2023; 2023. [Online; accessed 21 March 2023]. https://www.bcfi.be/nl/chapters/13?frag=20636.
3. The General practitioner: Price consultations in Belgium in 2023; 2023. [Online; accessed 21 March 2023]. https://www.cm.be/diensten-en-voordelen/ziekte-en-behandeling/terugbetalingen-behandelingen/huisartsen/raadplegingen.

4. Bilcke J, Marais C, Ogunjimi B, Willem L, Hens N, Beutels P. Cost-effectiveness of vaccination against herpes zoster in adults aged over 60 years in Belgium. Vaccine. 2012 Jan 11;30(3):675-84.
5. Bilcke J, Jan van Hoek A, Beutels P. Childhood varicella-zoster virus vaccination in Belgium: cost-effective only in the long run or without exogenous boosting?. Human vaccines & immunotherapeutics. 2013 Apr 1;9(4):812-22.
6. Bilcke J, Ogunjimi B, Marais C, De Smet F, Callens M, Callaert K, et al. The health and economic burden of chickenpox and herpes zoster in Belgium. Epidemiology & Infection. 2012;140(11):2096-109.
7. Pieters Z, Ogunjimi B, Beutels P, Bilcke J. Cost-effectiveness analysis of herpes zoster vaccination in 50-to 85-year-old immunocompetent Belgian cohorts: a comparison between no vaccination, the adjuvanted subunit vaccine, and live-attenuated vaccine. Pharmacoeconomics. 2022;40(4):461-76.
